# Calretinin and Parvalbumin Trapping of TDP43 and XRCC1 Instructs Neocortical Interneuron Death in Neonatal Hypoxic-Ischemic Encephalopathy

**DOI:** 10.3390/biom16050621

**Published:** 2026-04-22

**Authors:** Lee J. Martin, Rebecca N. Ichord, Caitlin E. O’Brien, Sophie Yohannan, Danay Fernandez, Annalise Garrido, Naya Amauri, Dongseok Park, Jordan Benderoth, Jennifer K. Lee

**Affiliations:** 1 Department of Pathology, Division of Neuropathology, Johns Hopkins University School of Medicine, Baltimore, MD 21205, USAdferna22@jhu.edu (D.F.); agarrid4@jh.edu (A.G.); eibrahi5@jh.edu (N.A.); dpark58@jhmi.edu (D.P.);; 2 Department of Neuroscience, Johns Hopkins University School of Medicine, Baltimore, MD 21205, USA; 3 Department of Anesthesiology and Critical Care Medicine, Johns Hopkins University School of Medicine, Baltimore, MD 21205, USA; 4 The Pathobiology Graduate Training Program, Johns Hopkins University School of Medicine, Baltimore, MD 21205, USA; 5 Department of Neurology, The Children’s Hospital of Philadelphia, Philadelphia, PA 19104, USA

**Keywords:** aggreosis, cell death, epilepsy, HIE, interneuron, protein nitration, seizure

## Abstract

We examined neocortical pathology and interneuron degeneration in neonatal hypoxia-ischemic encephalopathy (HIE). Piglets in two age groups (2–3 or 7–10 days old, n = 4–12/group) underwent global cerebral hypoxia–ischemia (HI) or sham treatment. Piglets (2–3 days old) had epidural electrodes for continuous electroencephalography (cEEG) and were treated with hypothermia (HT) or remained at normothermia (NT). Older piglets, all NT, had scalp EEG. Piglets at both ages had seizures and survived for 1–7 days. Cortical damage was assessed by hematoxylin & eosin staining and immunohistochemistry; calretinin (CR), parvalbumin (PV), and vasoactive intestinal peptide (VIP) interneurons (INs) were counted. Cell injury was assessed by DNA fragmentation and protein nitration. TAR DNA binding protein-43 (TDP43) and the DNA repair scaffold protein X-ray repair cross complementing-1 (XRCC1) were examined for degeneration mechanisms. Cortical layers 3 and 4 showed high vulnerability; damage emerged as isolated cells, focal and laminar, and distributed as panlaminar throughout different cortical regions that correlated with seizure burden. HT protected strongly against cortical damage. CR- and PV-INs were severely depleted in HI-NT piglets compared to sham. VIP INs appeared invulnerable. HT partially rescued the loss of INs. CR and PV formed nuclear and cytoplasmic inclusions that colocalized with TDP43 and XRCC1; co-immunoprecipitation identified interactions among these proteins, and tyrosine nitration of CR. CR and PV INs accumulated DNA single- and double-strand breaks and appeared as attritional apoptosis variants with proteinopathy. This cell death is identified as aggreosis. IN loss correlated with seizure presence. Postmortem human neonatal HIE cases had a similar loss of CR and PV INs and nuclear depletion of TDP43 in the neocortex. Thus, neonatal HIE causes the loss of neocortical inhibitory IN subtypes with vulnerabilities instructed by their intrinsic calcium-binding protein signature and by mechanisms consistent with toxic sequestration and the nuclear depletion of XRCC1 and TDP43 underlying DNA damage accumulation. Early inhibitory IN deletion could drive seizure evolution in HIE; TDP43 and XRCC1 could be therapeutic targets for neonatal HIE.

## 1. Introduction

Hypoxic–ischemic encephalopathy (HIE), caused by globally reduced brain oxygen and blood supply, is the leading cause of neurodevelopmental morbidity in term infants [1]. Term HIE infants develop bilateral injury to forebrain gray matter structures, including basal ganglia, the thalamus and peri-Rolandic neocortex [2,3]. Infants can also experience subacute refractory seizures in association with HIE, and these infants have increased mortality and a greater risk of long-term severe disability and epilepsy [4,5,6,7]. Though term newborns with moderate to severe HIE are treated with hypothermia (HT), infants still can have variably significant impairments in cognition years later and can develop cerebral palsy and persistent epilepsy. The use of HT has been contraindicated in low- to middle-income countries [8] and there is concerning evidence that rewarming might trigger seizures in some settings [9,10]. The effects of HT on subsequent seizure development in newborns are uncertain [9,11] and merit examination. The direct contribution of acute symptomatic seizures to the acute and delayed evolution of HIE in newborns has been debated for decades and remains understudied [12,13]. Moreover, existing treatment strategies for neonatal seizures have limited efficacy and unknown impact on the subsequent development of disability or chronic epilepsy [14] and are controversial [5]. All these uncertainties are partly due to an incomplete understanding of the cellular, molecular, and genetic mechanisms of HIE and associated seizures. Better experimental insight into the mechanisms of HIE-related seizures could be leveraged to identify novel targets (cells and biomolecules) and to develop new adjunctive therapeutics for HIE and neonatally acquired seizure disorders.

Interneuron (IN) involvement in the development of brain regional vulnerability and neurologic outcomes, including seizures, associated with neonatal HIE has been pursued for many years [15,16,17]. This rationale is sensible given the myriad subtypes (at least 25) of INs identified in animals and humans that regulate excitation/inhibition balance [18]. Experimental cell ablation studies have identified definitively that some INs prevent seizures [19]. Loss or degeneration of INs appears to occur in the hippocampus of postmortem human neonatal HIE cases [20], but events in neocortex are unknown. In experimental animal HIE using lissencephalic [21] and gyrencephalic [22,23] models, loss of INs has been reported. The loss of INs in a region identified as the parasagittal cortex has been seen in near-term fetal sheep [22,23,24], but these are not postnatal, freely moving, multisensory-world-experiencing, extrapersonal-space-integrating and responding animals [25]. Moreover, total cortical principal neuron loss and laminar damage or overall cortical damage and the mechanisms of IN loss are unclear in fetal sheep experiments. An understanding of IN subtypes related to principal neuron pools in the brain is important because INs can be lost secondary to the principal neurons they innervate [26,27].

We hypothesized that IN degeneration in HIE is related to their intrinsic calcium-binding proteins; they gain an abnormal property through nitration or oxidation that enables the sequestration of other proteins that sustain DNA and RNA integrity, thus causing single cell ablation. Of particular interest were TAR-DNA binding protein-43 (TDP43) and X-ray repair cross complementing-1 (XRCC1) because they function in cryptic exon suppression and DNA repair; somatic cell inactivation of both proteins is known to cause cell lethality [28,29], including IN death [30]. Because large animal models better represent the human neocortex compared to rodent models [31], we used a translational newborn piglet model of HIE that has been characterized extensively for its neuropathology [32,33,34] and as a model for associated seizure activity [32,35,36,37]. We then compared our findings in the hypoxia–ischemia (HI) piglet neocortex to postmortem human newborn HIE using the same IN markers to assess the clinical relevance of our animal model of neonatal brain injury.

## 2. Materials and Methods

### 2.1. Ethics Statement

All animal experimentation was conducted in accordance with the ARRIVE guidelines and the guidelines of the Helsinki Declaration and was approved by the Institutional Review Board of Johns Hopkins University (protocol number SW23M119, approval 6 June 2023).

### 2.2. Animals and Treatment Groups

Yorkshire piglets at two general ages (2–3 days and 7–10 days old) were used in randomized experimental designs. In these experiments, piglets were subjected to asphyxic cardiac arrest and cardiopulmonary resuscitation (CPR) to cause cerebral HI in survival models so that cellular and molecular neuropathology and post-ischemic seizures could be studied. The youngest neonatal piglets (2–3 days old, 1–2 kg, males) were used for neuroprotection experiments with hypothermia (HT) compared to normothermia (NT). The piglet treatment groups in this study were sham-normothermia (SH-NT, n = 6), sham-hypothermia (SH-HT, n = 10), HI-normothermia (HI-NT, n = 8), and HI-hypothermia (HI-HT, n = 10).

These piglets had continuous electroencephalogram (EEG) recordings with epidural electrodes and telemetry at the somatosensory cortices and survival for 2–7 days. Another cohort of piglets had HI at 2–3 days of age, but no EEG recording, and was euthanized at 29 h after the procedure to obtain unfixed freshly frozen brain tissue for western blotting and co-immunoprecipitation experiments; the group sizes for biochemical experiments were sham-NT (n = 4), sham-HT (n = 4), HI-NT (n = 4), HI-HT (n = 4), and naïve (n = 4).

Slightly older piglets (7–10 days old, 3.0–4.5 kg, males and females) were subjected to HI (n = 21) and sham (n = 6) procedures for neuropathology outcomes and scalp EEG recordings with survival of 4 days. Other piglets 7–10 days of age where subjected to HI or sham procedure and were survived for 24, 48, and 96 h for neuropathology studies (n= 6/time). We used 7–10-day-old piglets during an earlier period of our laboratory history when older animals were needed for better survival outcomes [32,35]. With improved model generation and critical care, and funding necessities requiring younger neonatal piglets, experiments were switched to 2–3-day-old piglets [34,36]. For both age groups, age-matched naïve piglets (n = 6/age range) were used for neurohistological comparators with sham piglets.

### 2.3. Asphyxic Cardiac Arrest in 7–10-Day-Old Piglets

Procedures for anesthesia, surgery and the asphyxic cardiac arrest protocol were described before [32,33,35]. Piglets were anesthetized with sodium pentobarbital 50 mg/kg ip, intubated and mechanically ventilated. Catheters were placed by sterile surgery in the descending aorta for hemodynamic monitoring and the inferior vena cava for drug delivery. Fentanyl 10 μg/kg iv and pancuronium 0.3 mg/kg iv were given for analgesia and muscular paralysis, respectively, with repeat treatments as needed (~every 1–2 h) for movement or pain responses prior to asphyxic arrest. Cephalothin 100 mg iv was given once daily throughout the protocol. Rectal temperature was maintained at NT (38.5–39.5 °C) using a heating blanket and overhead lamps. Intravenous 5% dextrose/normal saline was given (10 cc/h) to maintain normoglycemia (arterial glucose 60–90 mg/dL). During surgery and the asphyxic cardiac arrest protocol, piglets were monitored continuously for mean arterial pressure (MAP), heart rate, electrocardiogram (ECG), end-tidal CO_2_, fraction of inspired oxygen concentration (FiO_2_), and rectal temperature. Arterial blood gas, hemoglobin, O_2_ content, and glucose values were measured at regular intervals throughout the period of anesthesia.

After hemodynamic stabilization (about one hour after completion of surgery), hypoxia was induced for 30 min by adding nitrogen to the ventilator circuit and adjusting the FiO_2_ to 10–12% to achieve a partial pressure of arterial O_2_ (PaO_2_) between 20–25 mmHg while avoiding ischemic changes on the ECG. Afterwards, room air was restored to the ventilator circuit for 5 min to improve heart resuscitation. Asphyxia was induced by discontinuing mechanical ventilation and occluding the endotracheal tube for 7 min to induce cardiac arrest. CPR was performed by restoring mechanical ventilation with 100% FiO_2_, manual chest compressions, and epinephrine bolus 100 μg/kg iv. Bicarbonate 1 mEq/kg iv was given if MAP was <10 mmHg for 2 min or longer. Epinephrine and bicarbonate were repeated if needed at 3 min intervals until the restoration of spontaneous circulation (ROSC), defined as sinus rhythm with MAP > 60 mmHg. Additional bicarbonate was given after ROSC as needed until arterial pH was >7.30. After pH normalization and MAP stabilization > 60 mmHg for 30 min, ventilatory support was weaned over 2 h to pre-insult parameters. Sham piglets underwent the same surgery and anesthesia and post-operative care without exposure to hypoxia or airway occlusion.

### 2.4. Scalp EEG on 7–10-Day-Old Piglets

Prior to the asphyxia protocol, three bipolar channels of scalp EEG (left frontal-parietal, right frontal-parietal, posterior left-to-right parietal) were recorded with 0.5 cm gold cup Grass electrodes using a Grass EEG machine (Model 1020, Natus, Middleton, WI, USA) at a gain of 5 μV/mm, with filters set at 0.3 and 35 Hz, and digitized for continuous visual display and permanent electronic storage (Polyview, Grass EEG, Natus, Middleton WI, USA). Electrode impedance and voltage calibration were checked and recorded at the start of each day of recording. Visual surveillance of the piglet and EEG video display was performed continuously during recordings, which were annotated concerning the animal’s sleep state, clinical seizures, or motion artifact. EEG was recorded continuously from 30 min before hypoxia, through the asphyxic arrest protocol, and through 4–6 h after ROSC. On subsequent days, 30 min EEG recordings were obtained at 2–3-h intervals between 18 and 30 h after ROSC, and again at 2–3-h intervals between 36 and 50 h after ROSC, and at 6–8-h intervals thereafter to 96 h after ROSC, at which time the piglets were euthanized and perfused through a trans-myocardial catheter introduced into the ascending aorta with freshly prepared ice-cold 4% paraformaldehyde (PF) for tissue fixation and brain histology. One hemisphere was used for paraffin histology, and the other hemisphere was cryopreserved in 20% glycerol and frozen sectioned for immunohistochemistry.

### 2.5. Scalp EEG Analysis of 7–10-Day-Old HI and Sham Piglets

Digitized EEG recordings were visually analyzed offline in a manner blinded to the treatment group. EEG abnormalities were defined in HI piglets in comparison with normal patterns viewed in multiple archived EEG recordings from 10 naïve and sham piglets of the same age and strain undergoing similar anesthesia and recording techniques. The time from the onset of asphyxia to the appearance of isoelectric EEG was noted for each piglet. EEG recovery was graded according to the degree of discontinuity. For each piglet, the time after resuscitation at which each successive stage of recovery first appeared was noted. Seizure discharges were defined as rhythmic discharges showing evolution in amplitude, frequency, or morphology, and which do not appear in the EEG of normal piglets (Supplementary Figure S1). Seizure discharges were categorized as interictal (<10 s) or ictal (>10 s), and as either electrographic (no clinical seizure) or electroclinical (accompanied by clinical seizure activity). All seizure discharges in each recording were counted and characterized with respect to duration, localization, morphology, and frequency. A seizure severity index was calculated for each recording as the product of [median seizure duration X temporal density of seizure discharges], where temporal density = [100 × (sum of the durations of all seizure discharges/total duration of the recording)].

### 2.6. EEG, Asphyxic Cardiac Arrest, and HT with 2–3-Day-Old Piglets

Our HI-HT model using 2–3-day-old piglets has been described [34,36,37] and is summarized here (Supplementary Figure S2). Piglets were randomized into one of four experimental groups: sham-NT, sham-HT, HI-NT, or HI-HT (Supplementary Figure S2). Anesthesia was induced with isoflurane 5% and 50% nitrous oxide in 50% oxygen delivered by a nose cone. After intubation, the anesthetic was changed to 1.5–2% isoflurane and 70% nitrous oxide in 30% oxygen. Sterile catheters were placed in the external jugular vein and femoral artery. A fentanyl bolus (20 µg/kg iv) was given followed by 20 µg/kg/h. Additional fentanyl (10–20 µg/kg boluses) was given as needed for the management of apparent discomfort.

Epidural bipolar 4-lead continuous EEG (cEEG) electrode arrays (Stellar Telemetry, TSE Systems, Inc., Chesterfield, MO, USA) were installed into locations based on stereotaxic coordinates selected from the atlas of Salinas-Zeballos et al. [38], and our studies of piglet brain histology and connectivity [36]. Miniature cranial screws were inserted epidurally through burr holes, and the electrode–epidural screw assembly ([36], see Figure 1) was securely mounted with low-heat, quickset acrylic cement. The transmitter was inserted subdermal in a nape pocket, and the array antenna was secured to the posterior-most point of the scalp when it was sutured (Supplementary Video S1). cEEG recordings were analyzed as raw data and with post-acquisition processing (Supplementary Figure S3) as described [37].

After stable baseline cardiovascular and blood gas measurements were determined, inhaled O_2_ was decreased to 10% for 45 min causing a lowering of the PaO_2_ to ~30 mmHg and hemoglobin O_2_ saturation (SaO_2_) to ~30%. Then after 5 min of room air, required for successful cardiac resuscitation in this model, the piglets were made asphyxic (PaO_2_ ~15–18 mmHg and SaO_2_ ~3–5%) for 8 min by clamping the endotracheal tube to cause severe bradycardia (~50 bpm) and hypotension (~25–30 mmHg), followed by CPR using 50% inhaled oxygen, manual chest compressions, and epinephrine (100 µg/kg iv). The inhaled O_2_ was decreased to 30% after ROSC. Piglets that failed to regain consciousness within 3 min were excluded. Sham piglets received anesthesia, surgery, and inhaled 30% O_2_ but no hypoxia or cardiac arrest.

Whole-body HT was initiated 2 h after CPR or the sham procedure using ice packs and a water-circulating cooling blanket to a rectal temperature of 34 °C. This temperature reduction is used clinically in HT infants [39]. HT commenced 2 h after resuscitation to model a clinical delay in cooling patients. Ketamine (10 mg/kg/h iv) was initiated 3 h after resuscitation, and nitrous oxide was reduced. Dopamine was administered to maintain MAP above 40 mmHg during the overnight HT or NT protocols while the piglets were anesthetized.

Rewarming (0.5 °C/h) to NT began in HT piglets at 20 h from the onset by gradually increasing the temperature of the water circulating through the blanket. This rewarming rate is used clinically with infants. Piglets reached their NT target temperature of 38.5 °C ~29 h from onset. Vecuronium infusion was discontinued 29 h after the insult to allow neuromuscular blockade to dissipate before extubation. Piglets were extubated when they began to breathe voluntarily, regain muscle tone, were responsive to stimuli and established a tentative upright posture; then they were returned to cages with milk and overnight supervision.

### 2.7. Brain Harvesting for Neuropathology

The piglets survived for 1–7 days without anticonvulsant medications and were fed milk on a regular morning and afternoon schedule. Piglets that could not feed independently were euthanized. All brains were prepared for neuropathology with no postmortem delays. Euthanasia was performed with SomnaSol iv. Time-matched sham piglets were euthanized with HI piglets. A non-anesthetized unoperated cohort of piglets was a naive control group. cEEG electrode arrays were removed quickly from the skull before cardiorespiratory arrest from the SomnaSol. Piglets were perfused with phosphate-buffered saline for exsanguination and fixed with freshly prepared 4% PF. The piglet perfusion-fixation protocol was strictly followed as described previously [36]. After fixation, the animals were decapitated, and heads were immersed in 4% PF overnight. The following day the brain was removed carefully from the skull. Each brain was examined for electrode placement indicated by slight dural damage and for cortical damage. The protocol ensured cEEG screws were epidural without neocortical damage. Brains were then immersed in 20% glycerol.

The piglets that were triaged for fresh brain tissue harvesting were euthanized with SomnaSol iv and were quickly perfused with ice-cold PBS to flush the vasculature. The animals were decapitated, and the brains were rapidly extracted from the skull, cut into slabs with a chilled blade on a metal plate chilled by wet ice, and then microdissected into brain regions that were flash frozen in isopentane (−150 °C) and stored at −80 °C. These samples were homogenized and used for sodium dodecyl sulfate-polyacrylamide gel electrophoresis (SDS-PAGE)/western blotting for antibody validation with pig brain extracts and for co-immunoprecipitation to identify protein aberrant interactions after HI.

### 2.8. Neuropathology in Piglets with Injuries at 7–10 and 2–3 Days of Age Determined from Hematoxylin & Eosin (H&E)-Stained Brain Sections

Brains were blocked in the coronal plane in 5 mm slabs from the frontal pole to the occipital pole, and the slabs were placed in plastic cassettes for paraffin processing. Brain tissue paraffin bocks were cut into 10 µm thick sections using rotary microtomes. Floating paraffin sections were mounted from a heated water bath onto subbed glass microscope slides and dried on a warming tray. Sections were stained with H&E. All neuropathological assessments were performed blinded to treatment. Microscopic profile counting of total remaining normal and ischemic-necrotic neurons was performed at 1000× magnification in the primary somatosensory cortex of all piglet treatment groups as described [36]. This region was confirmed by cytology, chemo-architecture, and connectivity as previously described [32,36]. The primary somatosensory cortex has a known reproducible vulnerability in this model [32,34,35,36]. The piglets in the younger HI and sham cohorts were also used to determine total cortical damage from the frontal cortex to the occipital cortex in high-resolution digitally scanned images of anatomically matching H&E-stained sections from each piglet. For this quantitative method, all the neocortex was examined and segmented in digital images as histologically normal or damaged. The digital images were analyzed in the presence of the physical slide in the microscope for the real-time corroboration of damage or normality. Normal regions of the neocortex showed no evidence of ischemic–necrotic neurons, neuropil vacuolation, or glial swelling. Damaged regions of neocortex were identified by individual neuron necrosis, laminar damage, and panlaminar damage that were often seen as transitional gradients within the neocortex when inspecting different continuous gyri of the total cortical mantle.

### 2.9. IN Pathology and TDP43 Proteinopathy in Piglet Brain Determined by Immunohistochemistry

Single-label immunoperoxidase immunohistochemistry was used to identify INs and TDP43 localizations in the piglet brain. For the younger piglet cohort, paraffin section (10-µm) immunohistochemistry was performed. For the older cohort, paraffin sections and free-floating sections (40-µm) were used. For paraffin sections, citric acid buffer-heat antigen retrieval was used. Endogenous peroxidases were inactivated by methanol and H_2_O_2_. Normal goat serum was used as the blocking solution and Triton-x 100 was used as the detergent permeabilizer. The primary antibodies used were mouse monoclonal antibody (clone PARV19, Sigma-Aldrich, St. Louis MO, USA) and rabbit polyclonal antibody (abcam, ab11427, Waltham, MA, USA) to parvalbumin; mouse monoclonal antibody (clone 2D7A9, Proteintech, Rosemont, IL, USA) to calretinin; mouse monoclonal antibody (clone OTI1G1, LSBio, Newark, CA, USA) and rabbit polyclonal antibody (ImmunoStar, 20077, Hudson, WI, USA) to VIP; and rabbit polyclonal antibody to TDP43 (Proteintech). For negative controls, non-immune IgG isotype was used to replace the primary antibody at an identical µg/mL concentration. Affinity-purified goat anti-rabbit and goat anti-mouse secondary antibodies and corresponding peroxidase-antiperoxidase were used (Jackson ImmunoResearch Laboratories, West Grove, PA, USA). Diaminobenzidine (DAB) was the chromagen. Immunostained sections were sometimes counterstained with cresyl violet (CV) before coverslipping.

### 2.10. Dual Antigen Immunohistochemistry

To identify the co-localization of CR and PV with TDP43 and XRCC1, dual antigen detection was performed with DAB, cobalt/nickel-DAB, or benzidine dihydrochloride as chromogens as described [34]. Tissue injury and inflammation can result in considerable confounding autofluorescence, fluorescence over saturation in signals, and equivocation in antigen localization in degenerating neurons. To avoid these problems, we used immunoperoxidase double labeling with stringent inactivation of endogenous peroxidases. The double antigen localizations with these chromogens yield unequivocally contrasted colors under brightfield microscopy for single- and double-labeled cell identification [34]. CR mouse monoclonal antibody (Proteintech) was paired with TDP43 rabbit polyclonal antibody or with XRCC1 rabbit monoclonal antibody (Epitomics, clone EPR4389, Burlingame, CA, USA). PV mouse monoclonal antibody was paired with XRCC1 rabbit monoclonal antibody, or PV rabbit polyclonal antibody (Abcam) was paired with XRCC1 mouse monoclonal antibody (Invitrogen-ThermoFisher Scientific, clone 33-2-5, Waltham, MA, USA). For negative controls, non-immune IgG isotype was used to replace each pair of primary antibodies at identical µg/mL concentrations. CV counterstaining was not performed for immunohistochemical double-labeled sections.

### 2.11. DNA Damage Detection in Piglet Brain Sections

Different forms of DNA strand-breaks were localized in CR and PV INs. DNA double-strand breaks were detected with terminal deoxynucleotidyl transferase deoxyuridine triphosphate nick-end labeling (TUNEL). DNA single-strand breaks were detected with DNA polymerase I-mediated deoxyadenosine triphosphate nick-translation (PANT). These DNA strand-break detection strategies were combined with immunohistochemistry to detect DNA damage specifically in INs. Because both TUNEL and PANT require limited proteinase k digestion, IN markers were identified first, followed by DNA damage marking. Negative controls were non-immune IgG instead of primary antibody and the omission of terminal deoxynucleotidyl transferase or Klenow fragment of DNA polymerase I from the reaction.

### 2.12. Western Botting and Co-Immunoprecipitation

Frozen piglet brain samples were homogenized with a Brinkmann polytron in ice-cold 20 mM Tris HCl (pH 7.4) containing 10% (*w*/*v*) sucrose, 200 mM mannitol, complete protease inhibitor cocktail (Millipore-Sigma-Roche, St. Louis, MO, USA), 0.1 mM phenylmethylsulfonyl fluoride, 10 mM benzamidine, 1 mM EDTA, and 5 mM EGTA. Crude homogenates and cell lysates were sonicated for 15 s and then centrifuged at 1000× *g* for 10 min (4 °C). Protein concentrations were measured by bicinchoninic acid assay with a kit (Pierce, Thermo Scientific, Carlsbad, CA, USA) using bovine serum albumin as a standard. Piglet brain fractions and positive control cell lysates were subjected to SDS-PAGE and transferred to nitrocellulose membrane by electroelution. Ponceau S staining of nitrocellulose membranes before immunoblotting verified the lane equivalency of sample loading and transfer in each experiment. Blots of crude tissue and cell lysates were blocked with 2.5% nonfat dry milk with 0.1% Tween 20 in 50 mM Tris-buffered saline (pH 7.4), then incubated overnight at 4 °C with primary antibody to CR, PV, VIP, TDP43 or XRCC1. After the primary antibody incubation, blots were rinsed and then incubated with horseradish peroxidase-conjugated secondary antibody (0.2 µg/mL). Western blot negative controls were incubated with non-immune IgG isotype instead of primary antibody (Supplementary western blot file). For all blots, the primary and secondary antibodies were used at concentrations for visualizing protein immunoreactivity within the linear range. The blots were developed with an enhanced chemiluminescence (ECL) kit (Pierce) and imaged with a ChemiDoc imaging system (Bio-Rad, Hercules, CA, USA).

For immunoprecipitation (IP) prior to SDS-PAGE, piglet forebrain protein extract (500 µg) was input for 5 µg TDP43 or CR antibody followed by agarose-conjugated protein A (Pierce) for capture. Negative control conditions for IP experiments were homogenates from sham piglets, IP with isotype-specific non-immune IgG, IP with PBS instead of IgGs, and IP with no homogenate input. Final IP samples were subjected to SDS-PAGE and western blot using antibodies to TDP43, CR, or PV and ECL for detection.

### 2.13. Human Autopsy Brain Samples

Postmortem human brain samples were obtained from the Johns Hopkins University School of Medicine Brain Resource Center (Division of Neuropathology, Department of Pathology), the National Institute of Child Health and Development (NICHD) Brain and Tissue Bank for Developmental Disorders at the University of Maryland, Baltimore, MD, and the Children’s National Hospital, Washington, DC. All autopsies had approved consent, and tissues were de-identified. The human autopsy cases used are summarized (Supplementary Table S1) and have been characterized neuropathologically [34]. The protocols for using human autopsy tissue were reviewed and approved by the JHMI-IRB (NO:02-09024-04e, approved 27 September 2002) and the Children’s National Hospital IRB (IRB#15350, approved 22 December 2020, and #11850, approved 17 January 2019). The HIE cases received HT protocols prior to death or missed the therapeutic opportunity for HT (Supplementary Table S1). Infantile human non-HIE cases of spinal muscular atrophy or acute deaths due to non-neurological causes such as accidental death, pneumonia, or drug intoxication were used as comparators (Supplementary Table S1). Each human control case received an extensive microscopic review of 12–16 different brain region H&E sections to ensure the absence of neuropathology consistent with HIE and postmortem artifacts.

All human brains were immersion fixed in neutral-buffered formalin for at least 2 weeks before brain cutting. Paraffin-embedded brain sample blocks were cut into 7 µm sections on a rotary microtome. The sections were mounted on glass slides for immunoperoxidase immunohistochemistry with CR, PV, and TDP43 antibodies and detection with DAB. For negative controls, human brain sections were exposed to non-immune IgG isotype instead of the primary antibody at an identical µg/mL concentration.

### 2.14. Sample Size, Quantitative Data Presentation, and Statistical Analysis

Individual piglets were assigned to different treatments using a randomized study design. When making group side determinations, in prior neuropathology work on piglets the mean difference in damaged neurons within the neocortex between HI-NT and sham-NT piglets was 100, with a within-group standard deviation of 5 [34]. A sample size of four piglets generates power > 0.9. We increased the sample size to allow for some variability in our estimates.

**Figure 3:**
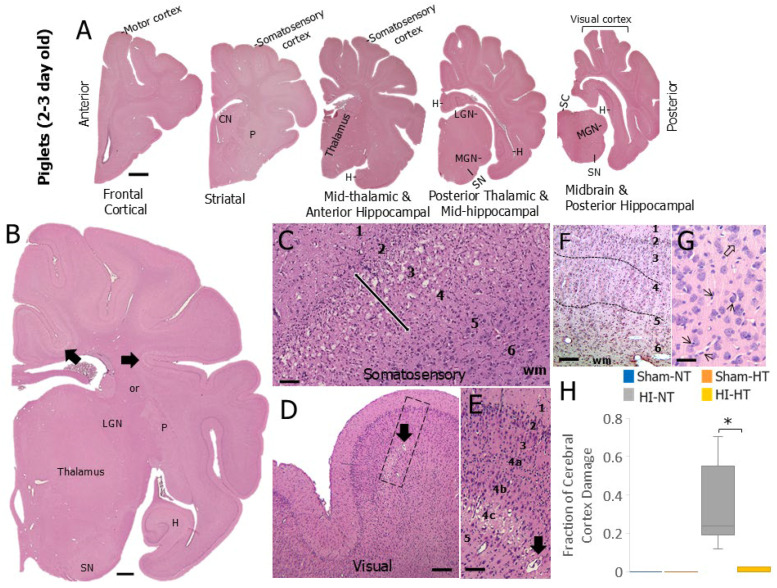
Neocortical pathology in neonatal piglets with HI at 2–3 days of age can be discretely laminar or pan-laminar depending on cortical region in the anterior-posterior neuraxis and is potently attenuated by HT. (**A**) The brains of piglets were systematically cut, stained with H&E, and examined for neuropathology from anterior frontal cortex to posterior visual cortex in the occipital region. Shown are representative H&E-stained sections of one piglet hemisphere from anterior (left) to posterior (right). The cortical and subcortical regions are identified. (**B**) Macro-image of an H&E-stained section from a brain of an HI-NT piglet that had EEG-identified seizures and laminar pathology seen as vacuolar and pale staining (black arrows) like that shown at higher magnification in panel (**C**). Identified brain regions are hippocampus (H), putamen (P), substantia nigra (SN), lateral geniculate nucleus (LGN), and optic radiation (or). (**C**) H&E staining showing the primary somatosensory cortex with the six layers identified to the subcortical white matter (wm) in a piglet with HI-NT treatment at 2–3-days of age. Damage to layers 2, 3, and 4 is prominent (double headed arrow) as evident by the loss of normal neurons and the vacuolar encephalopathy; neurons in layers 5 and 6 are mostly normal. (**D**) H&E-staining showing the visual cortex in a piglet with HI-NT treatment at 2–3-days of age. Selective laminar damage is seen (black arrow). (**E**) Boxed area in panel (**D**) showing the highly selective damage to layer 4c (a specific layer of primary visual cortex) seen as loss of normal neurons and the vacuolar encephalopathy; neurons in other layers are mostly normal. (**F**) H&E-staining showing a border zone in somatosensory cortex in a piglet with HI-NT treatment at 2–3-days of age. This pattern is identified as a border zone because it has accumulation of many small mononuclear inflammatory cells in the neuropil of layers 3 and 4, has neuronal pallor but without vacuolar neuropathology, and is confluent with an area of panlaminar damage. (**G**) High magnification of the layer 3–4 interface of a border zone showing a few hypochromic neurons (dashed arrows) and perivascular and perineuronal small mononuclear cells (thin arrows). (**H**) Box plot of median values (horizontal line) with Q1 and Q3 of the fraction of damaged neocortex in sham and HI piglets with or without overnight HT (n = 4–6 piglets/group). Asterisk indicates significant difference (*p* < 0.05). Scale bars: (**A**), 3 mm; (**B**), 1 mm; (**C**), 42 µm; (**D**), 500 µm; (**E**,**F**), 84 µm; (**G**), 21 µm.

**Figure 4:**
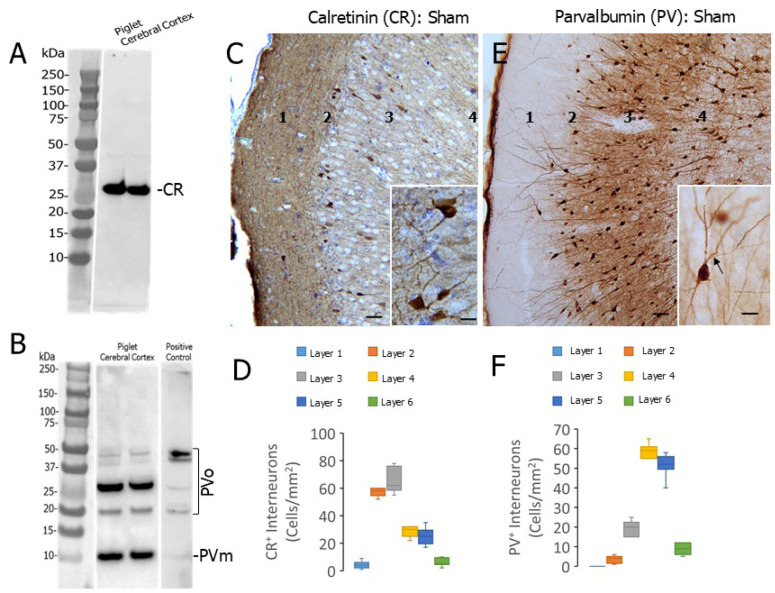
Normal localization of CR and PV INs in neonatal piglet somatosensory cortex. (**A**) Western blot of piglet neocortical extract showing the specificity of the CR antibody that was used for immunohistochemistry. Only one band that meets expectations for CR is detected. (**B**) Western blot of piglet neocortical extract showing the specificity of the PV antibody that was used for immunohistochemistry. A PV monomer band (PVm) was detected, but immunoreactive bands at higher molecular weights were also detected. HEK cell lysates overexpressing PV (positive control) also showed these immunoreactive bands suggesting that these higher bands in piglet band are PV oligomers (PVo). (**A**,**B**) Western blot original images can be found in Supplementary Materials. (**C**) Localization of CR immunoreactivity (brown staining) in the cortex of neonatal piglet. Layers 1–4 are identified. Inset shows higher magnification of immunoreactive cells (with CV counterstaining). (**D**). Box plot of median numbers with Q1 and Q3 (with the minimum and highest value whiskers) of CR INs in the different layers of cortex in sham piglets (n = 6). (**E**) Localization of PV immunoreactivity (brown staining) in the cortex of neonatal piglet. Layers 1–4 are identified. Inset shows higher magnification of immunoreactive cells. Arrow identifies a putative axon emerging from a PV cell body. (**F**) Box plot of median number (with IQR and the minimum and maximum value whiskers) of PV INs in the different layers of cortex in sham piglets (n = 6). Scale bars: (**C**,**E**), 30 µm; insets, 15 µm.

The data were analyzed using GraphPad Prism 9.5.1 or XLSTAT 2023.1.5 software. Data normality assessments were performed using the Shapiro–Wilk test. There was no exclusion of data points for any data set. All cell counting data was analyzed by one-way ANOVA and a post-hoc Holm–Sidak test. The use of ANOVA was justified because group treatment was categorically an independent variable and the variability of measurements within treatment groups appeared greater than the variability between treatment groups. The quantitative data in Figures 3H, 4D,F, 5B,E,G, 6B,C, 7B,C, 8C, 9G,H, 10E, 11D,E, 12E,F and 13E are illustrated as box-and-whisker plots. The plots show the data set with the minimum value (lower whisker), 1st quartile (Q1, 25th percentile, lower end of each box), median (the horizontal line in each box), 3rd quartile (Q3, 75th percentile, upper end of each box), and the maximum value (upper whisker). The spread of values in these graphical datasets is represented by showing the 25th and 75th percentile interquartile ranges (IQRs). The quantitative data in Figure 2C is shown as a standard histogram plot with the mean ± standard deviation (SD). Standard errors of the means were not used in the illustration of any dataset. *p* values < 0.05 were deemed statistically significant and are identified in the figures with an asterisk.

## 3. Results

### 3.1. EEG-Detected Seizures Develop in 7–10-Day-Old Piglets After HI with Severity Correlating with Neuropathology in Somatosensory Cortex

In 7–10-day-old piglets subjected to hypoxic–asphyxic cardiac arrest and CPR, the scalp EEG appeared unchanged during the hypoxia phase; but, within 2 min after the onset of asphyxia, the EEG became nearly isoelectric for >50% of the epochs with-low-voltage (5–10 µV) slow activity (1–5 Hz) (Supplementary Figure S1A). The recovery of background EEG activity with ROSC was temporally variable among piglets but generally progressed from isoelectric to discontinuous, and then continuous with mostly low-voltage (5–10 µV) slow and intermediate frequencies 1–8 Hz (Supplementary Figure S1D) succeeded by a pattern of continuous mixed voltages (10–50 µV) and a greater mix of 3–16 Hz frequencies (Supplementary Figure S1E). Within about 6 h after extubation, fully continuous EEG patterns emerged with mixed voltages and frequencies and spontaneous sleep–wake shifts (Supplementary Figure S1F).

Overlayed with this general pattern was a salient feature seen during the earliest phases of EEG recovery in about 50% of HI piglets typified by paroxysmal short runs of medium-to-high voltage (20–70 µV) bursting slow waves (Supplementary Figure S1C). Generally, these HI piglets had the slowest recovery of background EEG. These paroxysmal discharges never appeared in the EEGs of anesthetized sham piglets. There was a positive correlation between the duration of circulatory arrest (MAP < 10 mm Hg) and time to recovery of continuous EEG (R^2^ = 0.62, *p* = 0.001).

**Figure 1:**
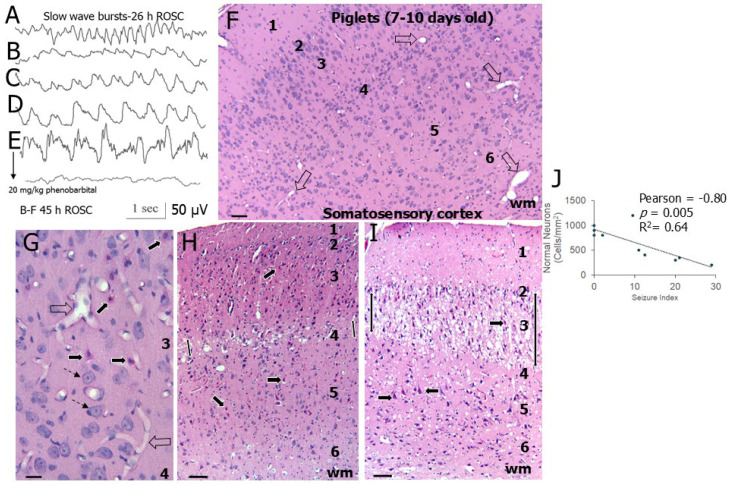
Typical seizure discharges in 7–10-day-old HI piglets and their neocortical neuropathology. (**A**) Rhythmic slow waves in a somnolent piglet at ~1 day (26 h) after ROSC. (**B**–**F**) Progression of a single seizure from its onset in “(**B**)” to its full development in “(**E**)” and then pharmacological termination 10 min later in a piglet at ~2 days (45 h) after ROSC. This EEG event (**A**) began during sleep without clinical symptoms initially and waxed and waned, but within minutes developed into a complex EEG identifiable seizure (**E**) and clinical seizure consisting of sudden arousal, repetitive rooting, and facial clonic movements (Supplementary Video S1). The lower tracing in “(**E**)” depicts the EEG at 10 min after administration of 20 mg/kg intravenous phenobarbital (arrow) in the same piglet. Seizure activity is resolved, and background EEG is suppressed. (**F**) H&E-staining of the primary somatosensory cortex. The six layers are identified to the deep subcortical white matter (wm) in a piglet with sham treatment at 7–10-days of age. Layer 4 is prominent. Open arrows identify patent small blood vessels free of residual blood and no perivascular swelling indicative of excellent perfusion-fixation. (**G**) HI piglet somatosensory cortex at 4 days after ROSC with isolated degenerating neurons with an eosinophilic cytoplasm and condensing basophilic nucleus (black-white highlighted arrows) and nearby morphologically normal neurons (dashed arrows) in layer 3. Open arrows identify patent small blood vessels. (**H**) HI piglet somatosensory cortex at 4 days after ROSC with very selective subtotal laminar vacuolar and perineuronal swelling (double headed arrows) at the layer 3 and layer 4 border and isolated degenerating neurons (black-white highlighted arrows). (**I**) HI piglet somatosensory cortex 4 days after ROSC with severe near-total laminar pathology in layer 3 and layer 2 (double headed arrows). This piglet had seizures. Individual degenerating neurons are identified (black–white highlighted arrows). (**J**) Loss of normal neurons in layer 3 correlated strongly with more severe seizures. Scale bars (µm): (**F**), 42; (**G**), 21; (**H**), 42; (**I**), 42.

EEG seizures were definitively seen in 7–10-day-old HI piglets at about 24–48 h after ROSC (Figure 1). Typically, seizure discharges emerged (Figure 1B–E) from rhythmic slow wave patterns in somnolent piglets (Figure 1A). These seizures manifested as an evolving pattern from rhythmic slow waves of increasing voltages (Figure 1C,D) to spike–wave complexes (Figure 1E) that also evolved in amplitude and frequency, sometimes reaching very high voltages (>100 μV) (Figure 1E). The identification of these waveforms as seizures was verified by their complete silencing by iv 20 mg/kg phenobarbital treatment (Figure 1F). Seizure discharges had their onset almost exclusively during sleep rather than during wakefulness and were bilateral and synchronous. Only a very small number of scalp EEG seizures were associated with clinically evident seizure activity.

Clinical seizures manifested predominantly as alterations in arousal and complex behavioral stereotypies, including repetitive snout twitching and rooting movements often progressing to facial clonic activity (Supplementary Material Videos S1–S4). Generalized clonic activity of the upper body could appear and then lower body and abrupt arousal from sleep could occur in an agitated state but with no upright posture. Clinical seizures were always preceded by escalating interictal seizure discharges which evolved into subclinical electrographic seizures, followed by frank electroclinical seizure activity.

These piglets were examined for neuropathology at 4 days of survival after HI (Figure 1F–I). The primary somatosensory cortex was evaluated because of its discernable laminar architecture with clear divisibility into six layers (Figure 1F) and known vulnerability and connectivity [36]. The severity of neuropathology within the somatosensory cortex varied in 7–10-day-old HI piglets; it ranged from isolated degenerating neurons in layer 3 (Figure 1G), selective laminar damage at the transition of layer 3 and 4 (Figure 1H), and more severe panlaminar damage manifesting in layers 2 through 4 with the epicenter in layer 3 (Figure 1I). Counts of remaining normal neurons in these layers revealed a significant loss correlating with seizure severity (Figure 1J).

### 3.2. EEG-Detected Seizures Develop in 2–3-Day-Old Piglets After HI Along with Global Neocortical Damage That Was Robustly Attenuated by HT

**Figure 2:**
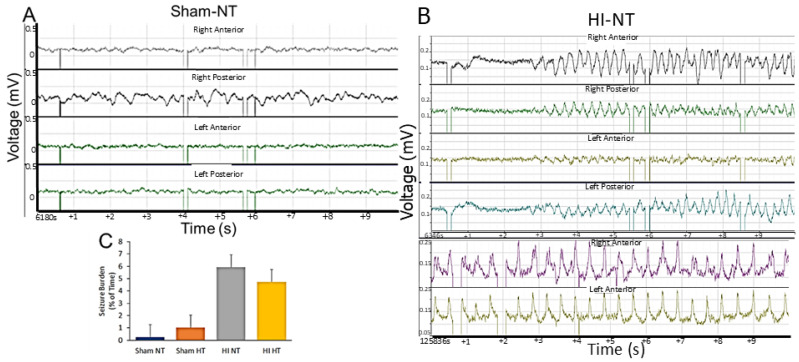
Epidural cEEG raw recordings from neonatal piglets with HI or sham treatments on 2–3 days of age and subacute recoveries under NT or HT. (**A**) Sham-NT piglet at ~3 h after extubation showing mostly low amplitude, high frequency (15–20 Hz) activity without epileptiform activity in most leads except for the right posterior lead that showed some bursting activity. (**B**) HI-NT piglet at ~3 h after extubation showing robust epileptiform activity. Frequent high-amplitude rhythmic slow spike–wave complexes emerged from periods of normal low-amplitude high-frequency activity and then developed (2 lower recordings) into higher amplitude spiking (200–250 µV). The epileptiform activity often started in the somatosensory cortex right anterior lead and quickly generalized to right posterior somatosensory cortex and then left anterior somatosensory cortex. (**C**) Seizure burden was calculated from the % of cEEG recording time showing epileptiform activity. Values are mean ± SD of n = 4–6 piglets/group. Seizure burden in HI-NT piglets nearly achieved significance compared to sham-NT piglets (*p* = 0.05).

Eighteen 2–3-day-old piglets had epidural cEEG and video monitoring after hypoxic-asphyxic cardiac arrest and CPR or sham procedure. cEEG revealed definitive seizures in this younger piglet cohort (Supplementary Videos S1–S4, Supplementary Figure S3). Sham piglet cEEG patterns showed activity of various frequencies, primarily high frequency (>10 Hz) and voltages of 10–100 µV, with variations in frequency and amplitude throughout all leads (Figure 2A). Fascinatingly different cortical areas in sham piglets had different baseline activities as evidenced by the right posterior cortex having higher amplitudes and different waveform patterns than the other areas of cortex (Figure 2A). Epileptiform patterns were generally divisible from baseline activity in HI piglets (Figure 2B, Supplementary Videos S1–S4). Seizures had antecedent high-amplitude and low-frequency activities (Figure 2B, top) that were focal and then evolved into rhythmic high amplitude spike–wave complexes (Figure 2B, bottom) primarily seen starting in the right anterior somatosensory cortex and then generalizing throughout cortical areas. These seizures could last 2–4 min before spontaneously resolving into baseline patterns. A few sham piglets in the NT and HT groups (these piglets were anesthetized for ~30 h) did show a low level of EEG seizure activity (Figure 2C). Overt clinical seizures were evident in some sham-HT piglets (Supplementary Video S6). The cEEG determined that the seizure burden tended to be higher in HI-NT piglets compared to sham-NT piglets (Figure 2C).

Clinical seizures usually emerged within 24 h after extubating during the second day after HI. Clinical seizures were often confirmed by real-time cEEG data where clinical seizure presence was directly linked to the ongoing cEEG acquisition (Supplementary Videos S1–S5). They generally appeared during sleep (Supplementary Videos S1–S5), consisting of orofacial twitching, tongue movements, rooting movement in the snout and head, repeated jerks of the head and legs, sudden arousal, and then clonic movements (see Supplementary Material Videos S1–S6). The clonic movements spread from the head region to the shoulder and forelegs to form generalized seizures (Supplementary Material Video S5). Some piglets developed fictive running movements when the seizure was believed to progress to status epilepticus (see Supplementary Material Videos S3 and S4). cEEG confirmed the presence of seizures with rhythmic spike–wave complexes of higher voltage (>400 µV) in specific neocortical areas that generalized to other areas of neocortex (Figure 3B, Supplemental Videos S1–S4). Some cEEG detected seizures appeared subclinical or equivocally clinical (Supplemental Videos S3 and S4).

The neocortex in 2–3-day-old neonatal piglets has very delineable regions, as shown previously using many different approaches [32,36,40] and here with H&E staining and immunohistochemistry for IN markers (Figure 3 and Figure 4). Generally, primary motor, primary somatosensory, and the primary visual cortex can be distinguished in the anterior–posterior axis in coronal sections (Figure 3A). Ischemic encephalopathy in some form was usually visible in the neocortex of HI piglets (Figure 3B). Like the 7–10-day-old piglets, damage in the 2–3-day-old piglet neocortex varied from selective laminar necrosis (Figure 3B–D) to panlaminar necrosis (Figure 3E). Moderate damage was seen as mostly laminar necrosis with neuropil vacuolar-spongiform pathology, typically affecting cortical layers 3 and 4 most prominently. Severe damage was seen as panlaminar necrosis, with ischemic–necrotic neurodegeneration and neuropil spongiform pathology spanning layers 1 through 5 in different cortical regions (Figure 3B,E). Isolated scattered neuron degeneration and small cell infiltration changes without neuropil vacuolation were discerned as the emergent, earliest, and mildest forms of cortical neuropathology appearing in layers 3 and 4 (Figure 3F,G). Prominent laminar pathology was always bordered by zones of small cell infiltration and neuropil pallor (Figure 3F,G).

These sections (Figure 3A) were used to quantify total cortical damage globally as indicated by tissue pallor and vacuolation at low magnification (Figure 3B), and higher magnifications were used to best discern cortical areas with more subtle damage (Figure 3D,F,G). The total fractional area of neocortex with damage was determined stereologically from the anterior-located frontal cortex to the posterior-located visual cortex (Figure 3A). The neocortex in 2–3-day-old HI-HT piglets had significantly less (*p* = 0.004) damage than HI-NT piglets (Figure 3H). Thus, HT profoundly protects the neocortex from HI damage in piglets.

### 3.3. CR and PV INs Have Discrete Laminar Distributions in Piglet Neocortex

Inspired by the discrete emergent isolated neuron and laminar distribution of neurodegeneration in the neocortex of HI piglets, we considered the localizations of IN subtypes in sham and HI piglets. We performed immunohistochemistry using an antibody to CR that specifically detected a protein of ~30 kDa in homogenates of piglet cerebral cortex (Figure 4A). The mobility of this protein on SDS-PAGE is consistent with CR in other animals [41,42]. We also used an antibody to PV for immunolocalization that detected a protein at ~11 kDa on western blots (Figure 4B), satisfying expectations for PV protein [43], and also putative SDS-stable oligomeric forms of PV in piglet cerebral cortex (Figure 4B). The immunoreactive bands at higher molecular weights than the predicted monomeric form of PV were uncertain until cell lysates overexpressing a PV cDNA were used for western blotting and probed with the same antibody that showed similarly migrating bands (Figure 4B, right lane). PV is known to form stable aggregates [43,44] that are likely detected in piglet cerebral cortex.

By immunohistochemistry, cells appearing morphologically as INs were CR-positive in all layers of piglet neocortex with a concentration in layers 2 and 3 (Figure 4C,D), and neuropil CR immunoreactivity was enriched in layer 1 (Figure 4C). PV-immunoreactivity had a contrasting localization with positive neurons concentrated in layers 4 and 5, low immunoreactivity in layer 1, and greater neuropil enrichment in deeper layers (Figure 4E,F).

### 3.4. PV and CR INs Die in Piglet Neocortex After HI, but VIP INs Remain Present

**Figure 5:**
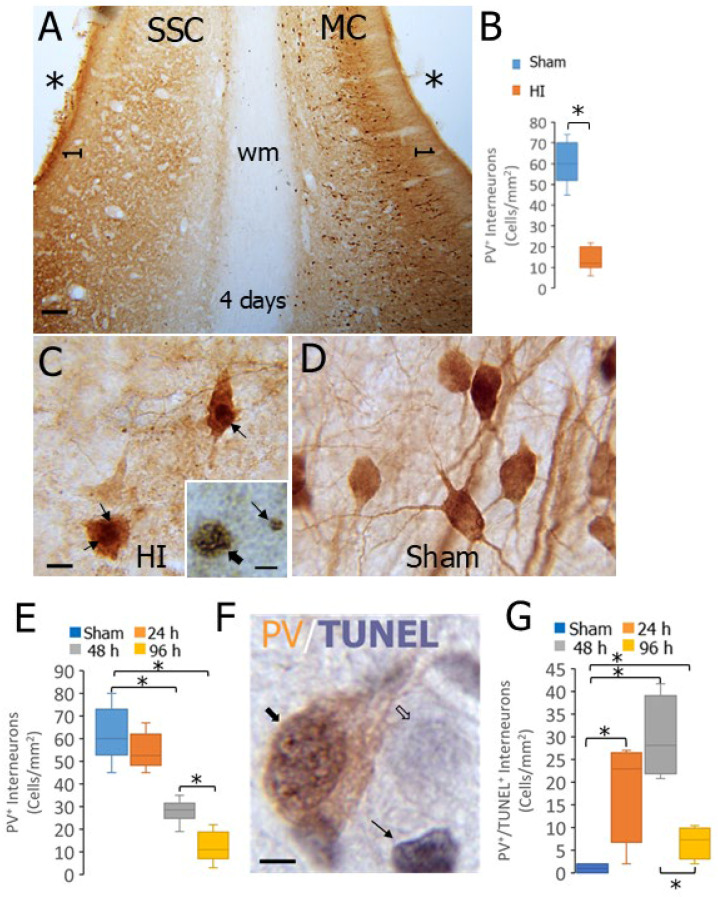
Profound degeneration and death of PV INs in the somatosensory cortex (SSC) of piglets with HI at 7–10 days of age. All asterisks in graphs indicate significant difference *p* < 0.05. (**A**) PV immunohistochemical staining reveals ablation of PV INs selectively in SSC but not in the motor cortex (MC) within the same gyrus at 4 days after HI and NT recovery. This piglet had clinical seizures. Layer 1 and subcortical white matter (wm) are identified. Asterisks identify the sulci on either side of the gyrus (medial is right). (**B**) Box plot of median numbers (with 25th and 75th percentiles and the minimum and maximum whiskers) of PV INs in the SSC in sham and HI piglets (n = 6/group). There is ~75% loss of PV INs 4 days after HI. (**C**) Residual PV INs after HI are attritional with strongly PV-positive nuclei and have aggregation and clumping of immunoreactivity (arrows). Inset shows degenerating PV IN with large PV-positive inclusions (broad arrow) and a PV IN fragment at the endstage of a cell death process identified as aggreosis (thin arrow). (**D**) PV INs in sham neocortex generally have open nuclei with light PV immunoreactivity. (**E**) Box plot of the median numbers (with IQRs and minimum and maximum value whiskers) of PV INs in the SSC in sham and HI piglets at 24, 48, and 96 h of NT recovery (n = 6/group). (**F**) PV INs accumulate DNA double-strand breaks (solid broad arrow). Nearby cells are either TUNEL-positive alone (thin arrow) or negative for PV and TUNEL (open arrow). (**G**) Box plot of the median numbers (with IQR and whiskers) of PV- and TUNEL-positive INs in the SSC in sham and HI piglets at 24, 48, and 96 h of NT recovery (n = 6/group). Scale bars (µm): (**A**), 176; (**C**) (same for (**D**)), 15; (**C**) inset, 14; (**F**), 5.

We examined the effect of HI on neocortical PV neurons in neonatal piglets. In 7–10-day-old piglets, PV-positive neurons appeared profoundly lost in the somatosensory cortex at 4 days after HI (Figure 5A, left), while PV neurons in the nearby motor cortex within the same gyrus appeared relatively preserved (Figure 5A, right). Counts of PV-positive neurons showed >80% loss of these neurons in the somatosensory cortex (Figure 5B, *p* < 0.0001). The remaining PV-positive neurons in HI piglets 4 days after the insult were severely attritional, with some appearing at the endstage of some form of shrinkage cell degeneration (Figure 5C), compared to the sham piglet cortex that contained PV neurons with robust cells bodies and elaborate dendrites in the neuropil (Figure 5D).

We then endeavored to identify the timing of the PV IN loss and whether these neurons indeed died as reflected by their genomic DNA fragmentation. Cohorts of piglets at 7–10 days old were subjected to HI and killed at 24, 48, and 96 h (Figure 5E). Though PV IN counts were slightly lower at 24 h compared to sham (Figure 5E), the first significant loss (*p* < 0.001) of PV INs occurred between 24–48 h after HI. The loss of PV INs was progressive, as shown by the additional significant decline in neuron number between 48 and 96 h (*p* < 0.001). To determine the likelihood that PV INs were dying, rather than simply losing their calcium-binding protein phenotype, we determine if they accumulated DNA double-strand breaks (DNA-DSBs) as assessed by TUNEL (Figure 5F). Though this method does not quantify the amount of DNA breakage, DNA-DSB accumulation is considered lethal to cells [45,46]. Subsets of PV-positive neurons were TUNEL-positive in HI piglets (Figure 5F). The numbers after HI were increased significantly (Figure 5G, *p* = 0.01) at 24 h compared to sham piglets and then further increased significantly (Figure 5G, *p* = 0.003) at 48 h compared to sham piglets. By 96 h after HI, the number of TUNEL-positive PV INs was significantly lower than at 48 h but still significantly greater than (*p* < 0.01) the sham value (Figure 5G).

**Figure 6:**
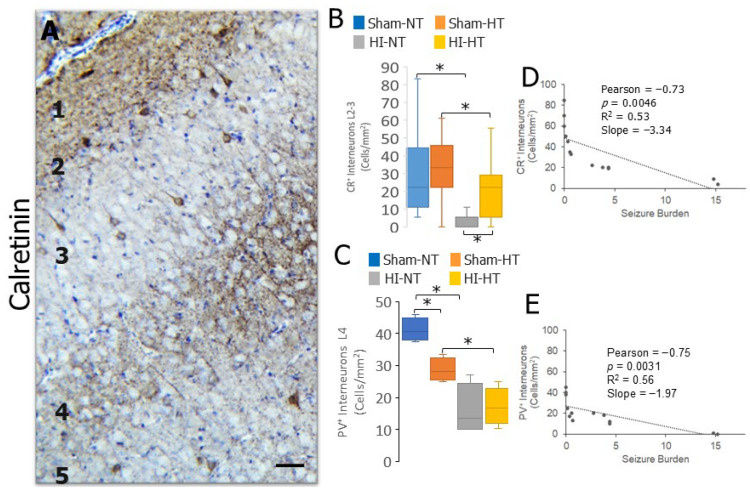
Depletion of CR and PV INs somatosensory cortex (SSC) correlates with seizure burden in piglets. All asterisks indicate significant difference *p* < 0.05. (**A**) CR immunoreactivity (brown) in SSC in layers 1–5 of a piglet after sham-HT procedures done at 2–3 days of age. Layers are identified at the left (blue is cresyl violet counterstain). Immunoreactive cell bodies, dendrites, and neuropil puncta are seen. (**B**) Box plot of median numbers (with 25th and 75th percentile ranges and minimum and maximum value whiskers) of CR INs in the SSC in piglets exposed to sham-NT (n = 4), sham-HT (n = 6), HI-NT (n = 4), or HI-HT (n = 4) procedures. (**C**) Box plot of the median numbers (with Q1 and Q3 IQRs and minimum and maximum value whiskers) of PV INs in the SSC in piglets exposed to sham-NT (n = 4), sham-HT (n = 6), HI-NT (n = 4), or HI-HT (n = 4) procedures. (**D**) Loss of CR INs significantly correlated with seizure burden in HI piglets. (**E**) Loss of PV INs significantly correlated with seizure burden in HI piglets. Scale bar (**A**) = 42 µm.

**Figure 7:**
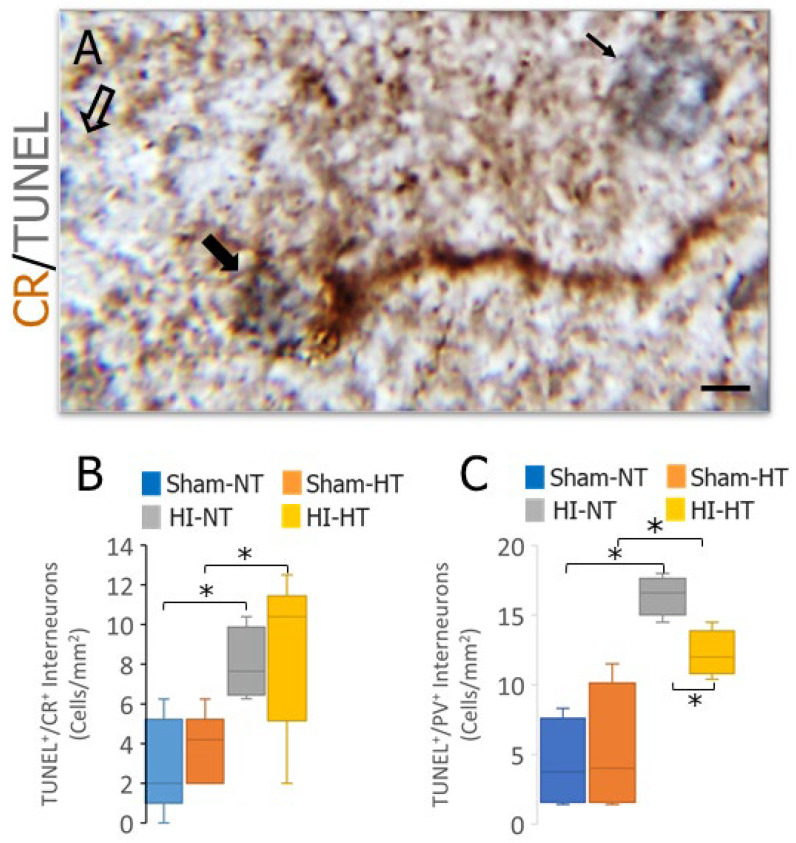
CR and PV INs die in the somatosensory cortex (SSC) of 2–3-day-old piglets exposed to HI and NT or HT recoveries. All asterisks indicate significant difference *p* < 0.05. (**A**) CR INs accumulate DNA double-strand breaks (solid broad arrow). Nearby cells are either TUNEL-positive alone (thin arrow) or negative for PV and TUNEL (open arrow). (**B**) Box plot of the median numbers (with 25th and 75th percentile IQRs and their minimum and maximum value whiskers) of CR- and TUNEL-positive INs in the SSC in piglets exposed to sham-NT (n = 4), sham-HT (n = 6), HI-NT (n = 4), or HI-HT (n = 4) procedures. (**C**) Box plot of the median numbers (with IQRs and whiskers) of PV- and TUNEL-positive INs in the SSC in piglets exposed to sham-NT (n = 4), sham-HT (n = 6), HI-NT (n = 4), or HI-HT (n = 4) procedures. Scale bar (**A**) = 4 µm.

The population of neocortical CR INs was examined to determine if the IN loss after HI in neonatal piglets was preferential for PV neurons. Moreover, we examined if body temperature management in piglets at clinically therapeutic HT levels [39,47] in sham and HI piglets affected the number of CR and PV INs. Like the PV INs, the CR IN cell bodies were a discrete population of neurons amenable to counting in 2–3-day-old piglet neocortex (Figure 6A). There was a significant loss of CR INs in HI-NT piglets compared to sham-NT piglets (Figure 6B, *p* < 0.0007). CR INs also accumulated DNA-DSBs after HI (Figure 7A,B, *p* = 0.005). Body cooling did not affect the number of neocortical CR INs in sham piglets (Figure 6B), and it did not cause the accumulation of DNA-DSBs in CR neurons in sham piglets (Figure 7B), but a partial rescue of the CR IN number was seen in HI-HT piglets compared to HI-NT piglets (Figure 6B, *p* < 0.003). HT in HI piglets also reduced the number of CR INs that accumulated DNA-DSBs compared to sham-HT piglets but not compared to the HI-NT group (Figure 7B). Body temperature management had unexpected effects in 2–3-day-old piglets. Cooling reduced the number of neocortical PV INs in sham piglets (Figure 6C, *p* = 0.001), but it did not cause the accumulation of DNA-DSBs in PV INs (Figure 7C). Cooling did not rescue the loss of PV IN number in HI-HT piglets compared to HI-NT piglets (Figure 6C), but it did reduce significantly the accumulation of DNA-DSBs in HI-HT piglets compared to HI-NT piglets (Figure 7C, *p* < 0.005).

We interrogated possible correlations among the neocortical IN number in the somatosensory cortex and seizure burden in 2–3-day-old HI piglets. The loss of both CR and PV INs significantly correlated with seizure burden, as indicated by Pearson’s correlation coefficients of −0.73 and −0.75, respectively (Figure 6D,E). The relationship between seizure burden and IN loss was stronger with CR INs, as indicated by the slopes.

**Figure 8:**
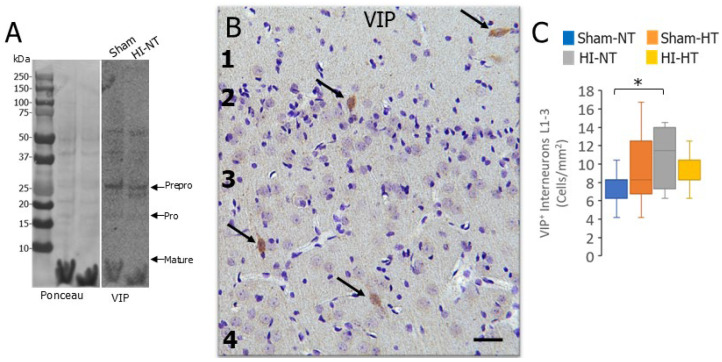
VIP INs in HI piglet somatosensory cortex (SSC) are spared. (**A**) Western blot of piglet neocortex extract showing the specificity of the VIP antibody that was used for immunohistochemistry. The detected bands meet expectations for the various forms of full-length, processed, and mature VIP. Western blot original images can be found in Supplementary Materials. (**B**) VIP immunoreactivity (brown) in layers 1–4 of a piglet SSC after sham procedures done at 2–3 days of age. Layers are identified at the left (blue is CV counterstain). Cell bodies (arrows) are seen. (**C**) Box plot of the median numbers (with 25th and 75th percentile ranges and their respective minimum and maximum value whiskers) of VIP-positive INs in the SSC of piglets exposed to sham-NT (n = 4), sham-HT (n = 6), HI-NT (n = 4), or HI-HT (n = 4) procedures. Asterisk indicates significant difference *p* < 0.05. Scale bar (**B**) = 32 µm.

We examined the VIP-positive subtype of IN in piglet neocortex to see it this neuronal type was vulnerable after HI like the PV and CR IN subtypes. By western blot screening, we identified an antibody that appeared to detect cleanly various full-length and processed forms of VIP in piglet cerebral cortex. Specifically, it detected putative prepro-, pro-, and mature VIP based on the molecular weight [48,49] of the identified bands (Figure 8A). By immunohistochemistry, this antibody detected subtypes of small IN-like cell bodies in the cerebral cortex, but the more numerous pyramidal neurons and glial cells were completely negative (Figure 8B). VIP-positive IN numbers were not significantly changed by HT in 2–3-day-old sham piglets, but in HI-NT piglets their numbers were significantly increased (*p* < 0.007) compared to sham-NT (Figure 8C).

### 3.5. Nuclear TDP43 Is Lost in Neurons in Neonatal Neocortex After HI

**Figure 9:**
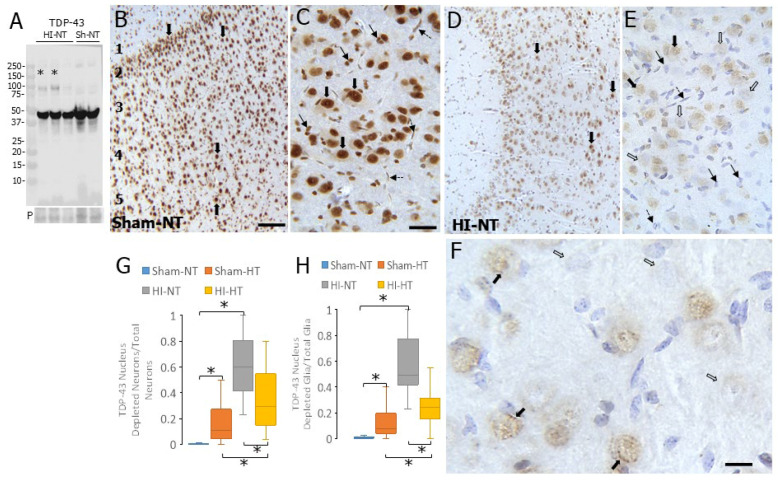
TDP43 is depleted in the somatosensory cortex (SSC) of piglets with HI at 2–3-days of age. All asterisks in graphs indicate significance difference *p* < 0.05. (**A**) Western blot of neocortex extracts from HI-NT and sham-NT (Sh-NT) piglets validating the specificity of the TDP43 antibody that was used for immunohistochemistry. Only one major band of immunoreactivity was detected; it met expectations for TDP43 based on size. A fainter higher molecular weight band (asterisks) was detected only in the HI piglets. Molecular weight markers are identified at left. Ponceau S staining of the membrane (P) shows protein loading. Western blot original images can be found in Supplementary Materials. (**B**) Localization of TDP43 immunoreactivity (brown staining) in the SSC of a neonatal piglet treated with sham-NT procedures. Layers 1–5 are identified at left. Immunoreactive cells (arrows) are ubiquitous. (**C**) Higher magnification image of sham-NT cortex showing the enrichment of TDP43 in the nucleus of neurons (broad arrows), glial cells (thin arrows), and endothelial cells (dashed arrow). (**D**) Localization of TDP43 immunoreactivity (brown staining) in the SSC of neonatal piglet treated with HI-NT procedures. Though some highly enriched neurons are present (arrows), overall staining is attenuated (sections shown in (**B**) and (**D**) were stained at the same time using identical solutions). TDP43 depleted cells are shown by the blue CV counterstain. (**E**) Higher magnification image of HI-NT cortex showing the prominent nuclear depletion of TDP43 in neurons (broad open arrows), glial cells (thin arrows), and other small cells (dashed arrow). Some neurons still have nuclear TDP43 (broad black arrows). (**F**) Some SSC neurons in HI-NT piglets show near complete nuclear and cytoplasmic depletion of TDP43 (open arrows). Intermingled neurons show depletion and TDP43 aggregate formation (solid black arrows). (**G**) Box plot of the median ratios (with Q1 and Q3 and respective minimum and maximum value whiskers) of neurons with nuclear TDP43 depletion to total neurons in the SSC in piglets exposed to sham-NT (n = 4), sham-HT (n = 6), HI-NT (n = 4), and HI-HT (n = 4) procedures. (**H**) Box plot of the median ratios (with IQR and whiskers) of glial cells with nuclear TDP43 depletion to total glial cells in the SSC in piglets exposed to sham-NT (n = 4), sham-HT (n = 6), HI-NT (n = 4), or HI-HT (n = 4) procedures. Scale bars (µm): (**B**,**D**), 100; (**C**,**E**), 28; (**F**), 11.

To begin examination of the upstream molecular mechanisms of IN loss in the neocortex of HI piglets, we turned our attention to TDP43 because it functions in DNA-DSB repair [50] and has been implicated specifically in CR and PV IN health and viability [51]. An antibody that cleanly detected TDP43, ascertained by molecular weight and abundance, on western blots of piglet cerebral cortex was identified (Figure 9A). Putative oligomeric forms of TDP43 were seen in HI piglets but not in sham piglets (Figure 9A). Oligomeric forms of pathological TDP43 have been identified [52]. We then used immunohistochemistry to localize TDP43 in the cerebral cortex of sham and HI piglets with and without body cooling. In sham-NT piglets, TDP43 was detected ubiquitously in virtually every cerebrocortical neuron, glial cell, and endothelial cell within all layers and was enriched prominently in the nucleus of these cells (Figure 9B,C,G,H). Overnight HT in sham piglets significantly reduced the number of neurons (*p* = 0.0003) and glial cells (*p* = 0.0004) with nuclear TDP43 compared to sham-NT piglets (Figure 9G,H). The nuclear TDP43 depletion was reduced even more strikingly in the neurons (*p* < 0.000001) and glial cells (*p* < 0.000001) of HI-NT piglet neocortex compared to sham-NT piglets (Figure 9F,G). The treatment of HI piglets with HT significantly mitigated the loss of nuclear TDP43 phenotype in neocortical neurons (*p* = 0.002) and glia (*p* = 0.0002) compared to HI-NT piglets (Figure 9G,H), but nuclear TDP43 in individual neurons (*p* = 0.008) and glia (*p* = 0.008) was still depleted compared to sham-HT (Figure 9G,H).

### 3.6. TDP43 Appears to Be Sequestered by Calcium-Binding Proteins CR and PV

**Figure 10:**
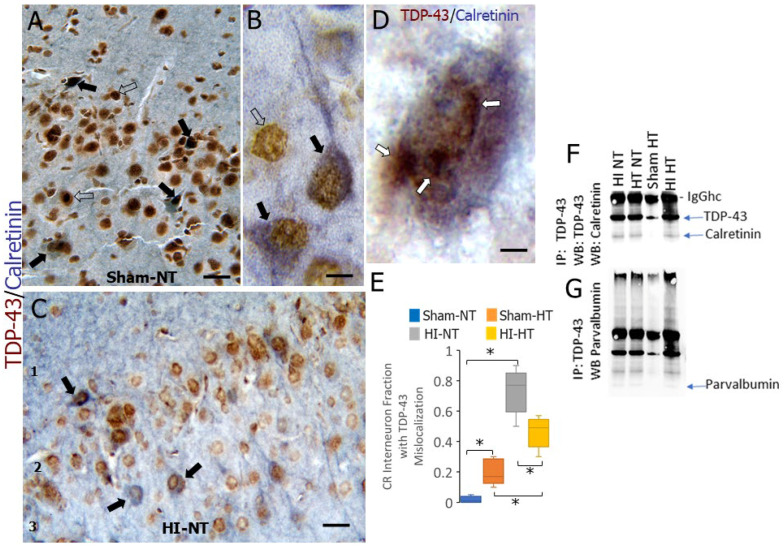
TDP43 appears trapped by CR and PV INs in somatosensory cortex (SSC) after HI in 2–3-day-old piglets. (**A**) Double labeling for TDP43 (brown) and CR (blue) in the SSC of a neonatal piglet treated with sham-NT procedures at 2–3 days of age. Layers 1, 2, and 3 are shown. TDP43 is enriched in virtually all cells. Subsets of neurons have nuclear TDP43 and cytoplasmic CR immunoreactivity (black broad arrows) indicating that they are INs. Other large cells (open arrows) have strong TDP43 nuclear labeling but not CR immunoreactivity indicating that they are not CR INs. (**B**) Higher magnification showing the clear identification of TDP43 in CR INs (black broad arrows) distinguishable for the TDP43 nuclear labeling in cells that are not CR-positive (open arrow). (**C**) Double labeling for TDP43 (brown) and CR (blue) in the SSC of a neonatal piglet treated with HI-NT procedures at 2–3 days of age. Layers 1, 2, and 3 are identified. Overall TDP43 immunoreactivity appears attenuated. Subsets of neurons have nuclear TDP43 and cytoplasmic CR immunoreactivity (black broad arrows). Many cells have partial nuclear clearance or redistribution of TDP43 immunoreactivity. TDP43 appears to be marginated to the nuclear membrane and associated with CR immunoreactivity. (**D**) Higher magnification of CR-positive IN showing the redistribution of TDP43 from the uniform nucleoplasmic enrichment to aggregations within the margins of the nucleus (white arrows) and into the cytoplasm in a pattern that overlaps with the CR immunoreactivity suggesting sequestration of TDP43 with CR. (**E**) Box plot of the median ratios (with IQRs and whiskers) of CR INs with nuclear TDP43 depletion to total CR INs in the SSC in piglets exposed to sham-NT (n = 4), sham-HT (n = 6), HI-NT (n = 4), or HI-HT (n = 4) procedures. Asterisks indicate significant difference *p* < 0.05. (**F**) TDP43 co-precipitates with CR in HT-NT and HT-HT piglet cortex but not in sham-HT cortex. TDP43 was immunoprecipitated (IP) followed by SDS-PAGE and WB for TDP43 and CR. Equivalent loading is shown by the IgG heavy chain (IgGhc). (**G**). TDP43 co-precipitates with PV in HT-NT and HT-HT piglet cortex but not in sham-HT cortex. The low molecular weight of PV (monomer at ~9–11 kDa) allows for reprobing of TDP-43/CR IP blots with antibody to PV to show interaction of TDP43 with PV in HI piglets. (**F**,**G**) Western blot original images can be found in Supplementary Materials. Scale bars (µm): (**A**), 22; (**B**), 11; (**C**), 5; (**D**), 22.

We localized TDP43 specifically in CR INs by dual-antigen labeling in the neocortex of piglets subjected to HI or sham procedures and temperature management at 2–3 days of age. In sham-NT piglets, TDP43 immunoreactivity was prominent in the nucleus, and CR immunoreactivity was prominent in the cytoplasm (Figure 10A,B). As anticipated from earlier results (Figure 9), an altered localization of TDP43 was observed in the neocortical neurons of HI piglets; specifically in CR INs, TDP43 was partially relocalized to the cytoplasm (Figure 10C,E) with the remaining nuclear TDP43 appearing aggregated and marginated near the nuclear membrane in combination with CR immunoreactivity (Figure 10D). HT itself caused the mislocalization of TDP43 in neocortical CR INs in sham piglets (Figure 10E, *p* = 0.0008) compared to sham-NT piglets. The most dramatically significant (*p* = 0.00001) mislocalization of TDP43 in CR INs occurred in HI-NT piglets compared to sham-NT piglets (Figure 10E). There was significant mitigation of the mislocalization in the HI-HT piglets compared to the HI-NT piglets (Figure 10E, *p* = 0.005), but the TDP43 mislocalization remained a significant phenotype in HI-HT piglets compared to sham-HT piglets (Figure 10E, *p* = 0.001).

The overlap and co-localization of immunoreactivities in brain tissue sections is insufficient evidence for the interaction and sequestration of proteins, so we used co-immunoprecipitation on freshly homogenized native non-denatured cerebral cortex tissue extracts of piglets. A reliable antibody for immunoprecipitating TDP43 was identified by immunoprecipitating TDP43, SDS-PAGE of captured proteins, and then western blotting for TDP43 (Figure 10F). CR, detected at ~30 kDa, co-precipitated with TDP43 in HI piglets (HT and NT) but not in sham piglets (Figure 10F). Similarly, PV, detected at ~11 kDa, co-precipitated with TDP43 in HI piglets but not in sham piglets (Figure 10G).

### 3.7. Neocortical INs in HI Piglets Appear to Sequester XRCC1 as Inclusions and Accumulate DNA Single-Strand Breaks (DNA-SSBs)

**Figure 11:**
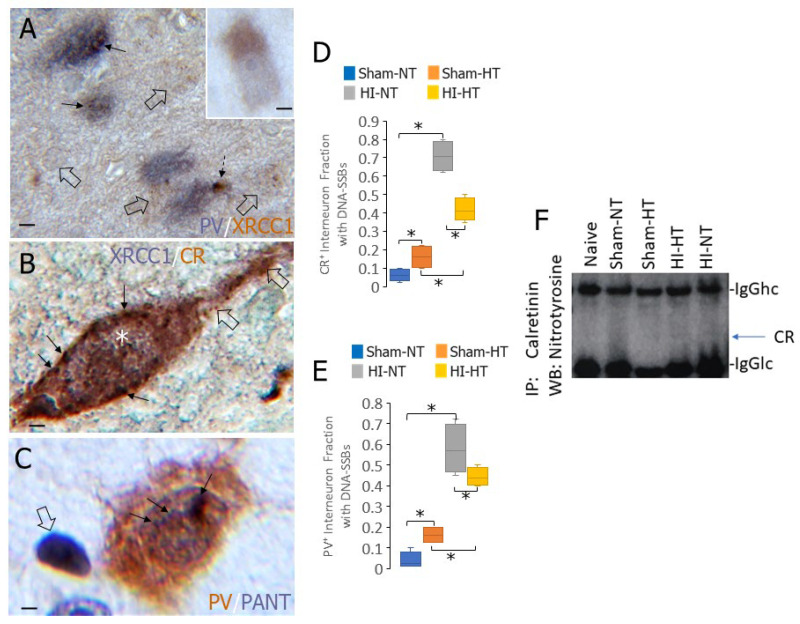
CR and PV INs in HI piglet somatosensory cortex (SSC) aggregate XRCC1 and accumulate DNA-SSBs. Asterisks in graphs indicate significance difference *p* < 0.05. (**A**) Double antigen immunohistochemical localization of PV (blue) and XRCC1 (brown) in 2–3-day-old sham piglet. XRCC1 appears as speckles and filaments within the nucleus of large cells likely to be neurons (open arrow) some of which are PV INs (black thin arrows). Inset shows a cortical PV IN in an HI-NT piglet with XRCC1 excluded from the nucleus. (**B**) XRCC1 (blue) is aggregated (black thin arrows) prominently in the perinuclear cytoplasm in CR neurons in HI-NT piglets. Asterisk identifies the cell nucleus. Single CR labeling (brown) is seen as boutons (open arrow) on the dendrite of this CR neuron. (**C**) PV IN (brown) in SSC of an HI-NT piglet with DNA-SSBs (blue, solid thin arrows) identified by PANT. Nearby is a cell nucleus enriched in DNA damage that is not PV-positive (open arrow). (**D**) Box plot of the median ratios (with 25th and 75th percentile ranges and minimum and maximum value whiskers) of CR INs with DNA-SSBs to total CR INs in the SSC in piglets exposed to sham-NT (n = 4), sham-HT (n = 6), HI-NT (n = 4), or HI-HT (n = 4) procedures. (**E**) Box plot of the median ratios (with Q1 and Q3 and their whiskers) of PV INs with DNA-SSBs to total PV INs in the SSC in piglets exposed to sham-NT (n = 4), sham-HT (n = 6), HI-NT (n = 4), and HI-HT (n = 4) procedures. (**F**) CR becomes tyrosine nitrated in HT-NT and HT-HT piglet cortex but not in sham-HT cortex. CR was immunoprecipitated (IP) followed by SDS-PAGE and western blotting (WB) for 3-nitrotyosine. Equivalent loading and usage of IgG is shown by the IgG heavy chain (IgGhc) and IgG light chain (IgGlc). Western blot original images can be found in Supplementary Materials. Scale bars (µm): (**A**), 7; (**A**) inset, (**B**,**C**), 2.5.

TDP43 is believed to function in nonhomologous end joining to repair DNA-DSBs [53,54]. We wondered if mechanisms for DNA-SSB repair were also altered in cortical INs in HI piglets. XRCC1 is a key scaffold protein that functions to detect nascent DNA-SSBs for targeted repair [55]. In normal sham piglet neurons, XRCC1 is localized to fine particles and granules throughout the nucleus, contrasting with the scant presence in the cytoplasm (Figure 11A). In PV and CR INs of HI piglets, XRCC1 was often localized to cytoplasmic and nuclear inclusions that colocalized with PV (Figure 11B) and CR (Figure 11C) immunoreactivities. The cytoplasmic sequestration of XRCC1 was very prominent in CR INs (Figure 11B).

DNA damage in neurons often involves the formation of DNA-SSBs that evolve into DNA-DSBs [56,57]. We thus used the PANT method, mediated by the Klenow fragment of DNA polymerase I [58], to determine if DNA-SSBs occur in neocortical INs of HI piglets. We assessed the presence of DNA-SSB in identified CR and PV INs (Figure 11C–E). Both CR and PV cortical INs in sham-NT piglets had scant detectable DNA-SSB (Figure 11D,C). HT in sham piglets significantly increased the accumulation of DNA-SSBs in CR (*p* = 0.01) and PV (*p* = 0.03) INs (Figure 11D,E). DNA-SSBs were markedly increased in CR (*p* < 0.0001) and PV (*p* < 0.00001) INs in HI-NT piglets (Figure 11D,E). HT significantly attenuated the accumulation of DNA-SSBs in CR and PV INs, but their accumulation remained significantly higher than in sham-HT piglets (Figure 11D,E).

### 3.8. CR Becomes Tyrosine Nitrated in HI Piglet Brain

The post-translational modification of proteins can cause normal proteins to gain aberrant properties. The nitration of tyrosine residues in proteins is one such modification that can drive cytotoxicity [59]. To explain the sequestration of TDP43 and XRCC1 by calcium-binding proteins, we examined the tyrosine nitration status of CR. Forebrain samples from naive, sham-NT, sham-HT, HI-HT, and HI-NT piglets were immunoprecipitated with a highly specific antibody to CR and then western blotted for 3-nitrotyrosine (Figure 11F). HI piglets, but not naïve or sham piglets, were found to have nitrated CR (Figure 11F).

### 3.9. CR and PV INs Are Lost in Human Neonatal HIE Neocortex

**Figure 12:**
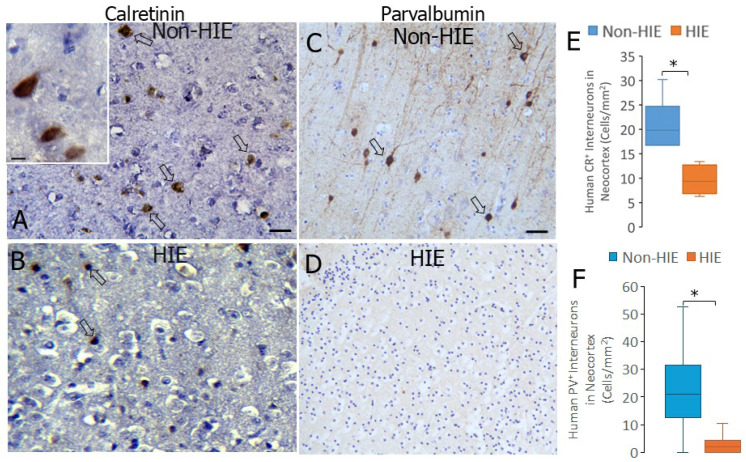
Loss of neocortical CR and PV INs occurs in human infant HIE. (**A**,**B**) Immunohistochemistry (with CV counterstaining seen as blue) for CR in non-HIE and HIE human parietal cortex identifies the population of CR-positive INs (open arrow, brown). Apparent partial depletion of CR IN number is seen. Inset shows control human CR INs at higher magnification. (**C**,**D**) Immunohistochemistry (with CV counterstaining seen as blue) for PV in non-HIE and HIE human parietal cortex identifies the population of CR-positive INs (open arrow, brown). Apparent depletion of PV INs is seen. (**E**) Box plot of the median numbers (with Q1 and Q3 percentiles and whiskers) of CR INs in the neocortex of human non-HIE (n = 5) and HIE (n = 6) cases. (**F**) Box plot of the median number (with IQRs and whiskers) of PV INs in the neocortex of human non-HIE (n = 5) and HIE (n = 6) cases. Asterisks in graphs indicate significant difference *p* < 0.05. Scale bars (µm): (**A**–**D**), 26; (**A**) inset, 13.

To ascertain the potential relevance of this work on neonatal piglet HIE in relation to human neonatal HI, we studied CR and PV INs in postmortem brains from term infant newborns that died from HIE compared to newborns suffering from non-HIE-related deaths (Figure 12). CR (Figure 12A) and PV (Figure 12C) immunoreactivities were localized to discrete non-pyramidal neurons in human newborn neocortex. These neurons were very amenable to counting. Neonatal HIE brains showed a significant loss of CR (*p* = 0.004) and PV (*p* < 0.0001) INs compared to non-HIE cases (Figure 12E,F).

### 3.10. Nuclear TDP43 Is Depleted in Neocortical Neurons in Human Neonatal HIE

**Figure 13:**
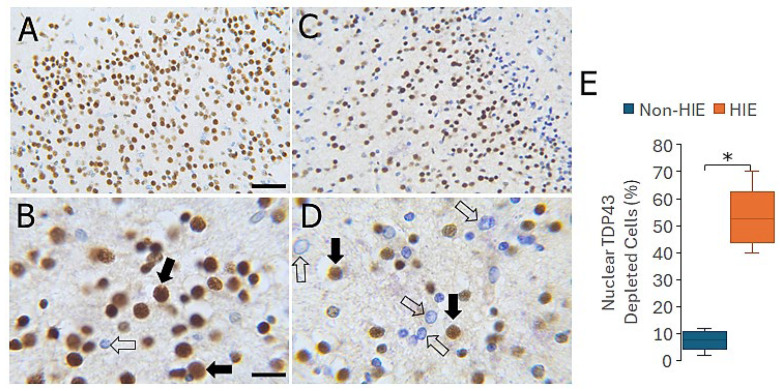
Nuclear TDP43 is depleted in cerebral cortex of human HIE. Blue CV counterstain shows total cells. (**A**,**B**) Localization of TDP43 immunoreactivity (brown) in non-HIE parietal cortex. Most cells have strong nuclear staining ((**B**), solid black arrows); very few cells are negative as seen by the CV with no brown ((**B**), open arrow). (**C**,**D**) Localization of TDP43 immunoreactivity (brown) in HIE parietal cortex. TDP43-positive nuclei are present ((**D**), solid black arrows) but many cells have lost TDP43 immunoreactivity ((**D**), open arrows). (**E**) Box plot of the median percentage (with Q1 and Q3 and their minimum and maximum value whiskers, respectively) of cells with nuclear TDP43 depletion in the neocortex of human non-HIE (n = 5) and HIE (n = 6) cases. Asterisk indicates *p* < 0.05. Scale bars (µm): (**A**,**C**), 80; (**B**,**D**), 27.

We immunostained human postmortem brain sections for TDP43 (Figure 13A–D) to determine if there was a loss of nuclear TDP43 like that seen in the HI piglet neocortex. Infant HIE brains showed a significant increase (*p* < 0.001) in the number of neurons in the parietal cortex without nuclear TDP43 (Figure 13E).

## 4. Discussion

**Figure 14:**
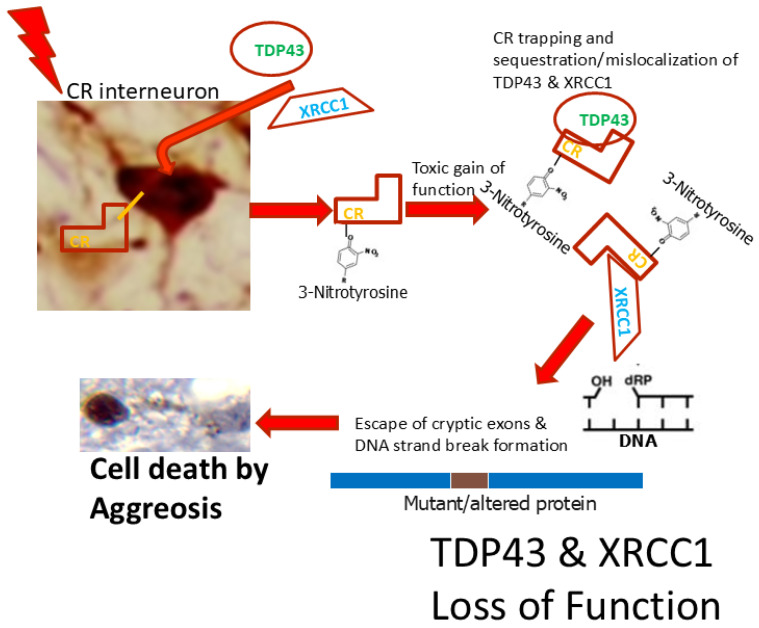
Evidenced-based hypothetical summary of the mechanisms of neocortical IN degeneration in neonatal HIE. The IN degeneration is subtype selective and laminar. INs could develop TDP43 and XRCC1 loss-of-function by trapping/sequestration mechanisms imparted by their inherent calcium-binding proteins that gain an aberrant property after nitrative stress and protein 3-nitrotyrosine formation or oxidative damage. TDP43 and XRCC1 loss of function could lead to single cell DNA damage and strand break formation or TDP43 failure to suppress cryptic exons (brown domain within blue protein structure schematic in lower right). We name this cell death form aggreosis.

This work is a novel and important contribution to neonatal encephalopathy. We studied neocortical INs and their loss in experimental and clinical HIE. We show that piglets subjected to asphyxic cardiac arrest, to cause cerebral HI, and CPR at 2–3 and 7–10 days of age have IN loss that is like that seen in postmortem brains of human infants that died from HIE. Our piglet model is prepared optimally for the cellular and molecular interrogation of neuropathological mechanisms, and the information gleaned is likely to be meaningful and translationally relevant. In piglets, INs manifested DNA damage accumulation within 24 h after the insult, and a significant loss of INs was first seen between 24–48 h after the insult. This timing is consistent with the neurodegeneration seen in pure DNA damage-induced cell death paradigms in cell culture [60]. The degeneration and loss of INs persisted through at least 4 days after the clinical insult. CR and PV INs, but not VIP INs, were vulnerable. The neocortical damage in general and the specific loss of PV and CR INs correlated with seizure burden during recovery. The death of INs involved the accumulation of unresolved DNA-SSBs and -DSBs. The DNA damage accumulation appears related to the loss of function of TDP43 and XRCC1 caused by the sequestration and aggregation of these two DNA repair proteins in a cell death process we describe as aggreosis (Figure 14). Nitration or oxidation of the signature calcium-binding proteins of these INs could explain both the mechanism of their degeneration and their intrinsic vulnerability to somatic cell conditional ablation. These experiments identify a novel and intriguing cellular pathology in INs found in neonatal HIE that could be a molecular mechanism involved in neurodegeneration and seizure origin in infants with acquired brain injury. TDP43 and XRCC1 rescue of DNA repair function could be therapeutic targets for improved recovery of neonates with HIE. Restoration of DNA repair has been identified previously as a realistic therapeutic target for neuroprotection in vivo [61].

### 4.1. EEG-Confirmed Seizure Activity After HI Associates with Worsened Neocortical Damage That Can Be Attenuated by HT

We used piglets at different neonatal age ranges (2–3 and 7–10 days) to study seizure activity, neocortical pathology, and IN loss. The younger piglets were also used to assess the therapeutic efficacy of HT on neocortical damage and IN loss. Two different general ages of piglets were used because INs undergo complex spatiotemporal developmental changes in this gyrencephalic large animal in short times [62] that could impact vulnerability in our brain injury model. In addition, in the history of our laboratory, we have transitioned from older piglets [32,33,35] to younger newborn piglets [36], and scalp EEG [35] to more intensive epidural cEEG with telemetry [36,37] for grant funding needs. Despite age differences, seizures and IN loss were seen in both groups that correspond roughly to the human brain at birth and in infancy [62].

We observed in 7–10-day-old piglets EEG waveforms after HI that could predict later seizure activity in correlation with the severity of neocortical pathology. Specifically, abnormalities in the recovery of EEG during the first few hours after resuscitation predict the severity of neuropathology and clinical outcome; moreover, transient cortical hyperexcitability seen on EEG early after HI could be signatory of evolving neocortical damage. This interpretation harmonizes with a previous study demonstrating that early recovery of EEG burst counts and duration are prognostic for outcome at 24 h after the insult [63]. Earlier bursting precedes better outcome. These findings suggest that HI insults to the neonatal brain, severe enough to cause neocortical neurodegeneration, involve mechanisms in which the earliest phase is associated with a silencing of synaptic transmission, evidenced by isoelectric EEG and the slow recovery of continuous EEG activity, followed by a transient imbalance between excitatory and inhibitory systems that manifest as electrographic seizure discharges. Panlaminar neuropathology in primary somatosensory closely correlated with seizure severity.

Our epidural cEEG revealed regionality to the evolution of abnormal neocortical electrophysiology in the neonatal brain after HI in the younger piglets (2–3 days old). This approach uncovered much more about the anterior–posterior and right–left hemisphere differences in the somatosensory cortex in relation to seizure evolution than scalp EEG. Among the bilateral recording electrodes in the anterior and posterior regions of the somatosensory cortex, the right anterior primary somatosensory cortex appeared to be a focus of epileptogenesis, with generalization of seizure activity to the left hemisphere and then involving more posterior regions. Interestingly, this seizure generalization pattern matches the involvement of neocortical layer 3 in corticocortical feedforward connectivity [64] and the predictable emergence of neuropathology earliest in deep layer 3 or in upper layer 4. Several HI piglets with NT recovery developed status epilepticus and required urgent euthanasia and perfusion–fixation. This was done without delay owing to our 24/7 supervision of the animals. The neocortex was globally damaged in these piglets. Mild (38.5 → 34.5 °C) whole-body HT for 29 h during recovery robustly protected against this global damage to the neocortex in general and specifically within the primary somatosensory cortex. Mild HT has been shown before to be neuroprotective in the neocortex in neonatal pigs [65] and fetal sheep [66]. These earlier studies showed neuroprotection in a limited region of unspecified parasagittal neocortex. We show here the global neocortical protection afforded by HT.

The primary somatosensory neocortex in piglets is selectively more vulnerable to the effects of O_2_ and energy substrate deprivation than other cortical regions in the absence of seizures [32]. Our model matches the peri-Rolandic vulnerability in human infants with HIE [3]. This vulnerability might be specified by its higher baseline oxidative metabolism, its connectivity, and the laminar and neuronal architecture [32,36]. Brainstem sensory relay centers have higher cerebrovascular prefusion than the cerebral cortex in piglet; yet the brainstem is functionally preserved compared to the neocortex under conditions of severe partial ischemia [67]. Brainstem neurons lack spines and are isodendritic compared to the neocortex with spine laden pyramidal neurons [68,69]. The relative insensitivity of the brainstem to HI would allow for the persistence of ascending sensory inputs to activate the ventral posterior thalamus that drives primary somatosensory cortex layer 4 [70]. Thalamic afferents are associated with the direct and potent excitatory drive of INs in layer 4 [71] at a time when neocortical neurons are challenged by anoxic depolarization or are in a state of diminished recovery. Brainstem and thalamic afferents can also heighten cortical excitability [72]. The greater the delay in the recovery of energy metabolism and membrane potential, the more prolonged the period of anoxic depolarization, and the greater the likelihood of irreversible damage to neocortical neuron dendrites from unregulated Na^+^ and Ca^2+^ influx and excitotoxic levels of glutamate. This pathological progression is supported by evidence for NMDA receptor activation in HI piglet forebrain [73,74]. The early pathological stages could be reflected by small cell inflammatory changes at border zones of frank injury in the transitional regions of neocortical layers 3 and 4. Lasting impairments in mitochondrial ATP production, as measured by complex IV activity, and cell volume control enzymes such as Na^+^/K^+^-ATPase activity are prominent characteristics of subacute evolving neurodegeneration in these regions of neocortex most susceptible to damage in this model [31,36]. The robust protection afforded to the neocortex by overnight HT, even implemented after a 2 h delay, suggests that the subacute recovery (6–12 h after HI) is critical for neuropathology evolution.

### 4.2. EEG-Confirmed Seizure Activity After HI Associates with IN Loss That Is Rescued by HT

Struck by the reproducible laminar pathology that first emerges in layer 3 and upper layer 4 of the somatosensory cortex and by the seizures that manifest in this region subacutely about 24 h after HI, we thought of inhibitory IN loss of function, in addition to the more overt degeneration of pyramidal neurons. A single pyramidal neuron receives thousands of excitatory and inhibitory synapses [69,75,76]. Many inhibitory synapses in neocortex are derived from the elaborate variety of IN subtypes [77,78,79]. Calcium-binding proteins are excellent for identifying the cell bodies, dendritic arbors, and presynaptic terminals of some IN subtypes [80,81]. However, scant information is available on INs in the pig brain. Two studies have reported CR and PV INs in naïve newborn and adult pigs [82,83]. Our study advances prior work with a quantitative analysis of CR and PV in different layers of the somatosensory cortex of sham and HI piglets. The antibodies used were highly specific with pig brain tissue, as shown by western blotting. Antibody specificity always needs to be validated for immunohistochemical applications in each species of animal [84]. This was not done in other studies of large animal models of HI and seizures [22,23,24]. We found that PV was prone to forming stable aggregates with heat treatment [44,80], but CR did not appear aggregation prone. The neurons identified by immunohistochemistry were exquisitely delineated. The laminar distributions of CR and PV INs were different. CR neurons were concentrated in layers 2 and 3. PV neurons were concentrated in layers 4 and 5. These patterns fit with our hypothesis of IN vulnerability to HI and possible involvement in HIE-related seizure evolution.

The cortical regional vulnerability of calcium-binding protein containing INs in the somatosensory cortex was striking in HI piglets. There was ~80% depletion of PV INs by 4 days. Their death started by 1 day after HI, as marked by TUNEL, which shows the accumulation of DNA-DSBs [85]. CR INs were also vulnerable in HI piglets. Cortical VIP-positive INs were not lost in HI piglets; rather, this population of INs was significantly increased HI-NT piglets. CR INs were partially protected by HT, but PV INs were not protected by HT. The insensitivity of PV INs to cooling in HI piglets could be related to the inherent ability of PV to aggregate, as shown by our western blots. The loss of INs correlated significantly with seizure burden. The correlation was more robust with PV IN loss. However, our study does not establish causal links between IN loss and seizures. Causality determination will require complex genetic studies in pigs. Our cEEG data and neuropathology localization suggest that seizure could emerge focally within a specific area, and perhaps even a specific layer, of the somatosensory neocortex. Conditional gene-targeting strategies using adeno-associated virus serotype 9 for TDP43 or XRCC1 rescue of function within specific IN populations driven by cell type-specific promoters within somatosensory cortex would be an experimental paradigm able to test IN and brain region causality and the molecular mechanism of seizure origin.

Few studies of neocortical INs in the gyrencephalic brain after HI are available. Most work is from one lab that used fetal sheep. In an undefined area of the parasagittal cortex and undefined layers, the loss of glutamic acid decarboxylase- and PV-positive neurons and perineuronal nets was shown [22,23]. The loss of INs generally correlated with seizure burden, as determined by epidural EEG [22], and was attenuated by HT [24]. These results in fetal sheep mirror ours in neonatal piglets. However, our study in piglets has important differences from the previous work in fetal sheep. We studied the postnatal brain, specifically in the primary somatosensory cortex characterized previously [32,36,40] and in specific layers of the cortex identified, using antibodies validated using western blotting with swine brain extracts, to have the highest concentrations of these particular INs. We identified an invulnerable population of VIP-positive INs. We confirmed by DNA damage detection that the INs were dying, ruling out that they were merely losing their phenotype. The loss of neurochemical phenotype without cell death can occur in other settings of acquired brain injury [86]. We determined the time course of IN death with DNA damage accumulation emerging before 24 h and then the significant loss of INs beginning between 24–48 h. We also identified a possible novel molecular mechanism for their death and an explanation for IN vulnerability that is instructed by their intrinsic calcium-binding protein signature. A prior study of adult rat brain ischemia also suggested that calcium-binding proteins might predispose subtypes of neurons to death [87].

### 4.3. IN Loss Occurs in Human HIE Neocortex

INs account for 10–15% of the cortical neurons in rodents [78] and 20–30% of cortical neurons in humans [88]. While some aspects of INs are conserved among different species, differences among INs in different animals are also prominent [69,88]. For example, there is a 2.5-fold increase in inhibitory INs from mice to humans, and the highly integrative IN-to-IN network seen in the human brain is virtually absent in the mouse brain [79]. Moreover, human neurons in cell culture engage widely different cell stress, DNA damage repair, and cell death responses compared to mouse neurons [89]. We therefore uniquely examined the translational relevance of our findings in HI piglets by a comparison of the same IN types in human HIE. Sham piglets had at least double the number of CR and PV INs in layers 3 and 4 of the neocortex compared to the non-HIE human brain. However, this finding is based on profile counting. Stereological counting methods need to be used in the future. In the human HIE brain, both CR and PV cells were depleted. The PV INs were more affected in the human brain than the CR INs. These results in human HIE postmortem brains are unlikely to be related to postmortem artifacts because similar results were seen in our piglet models where the brains are prepared by perfusion–fixation with exceptionally high quality. Similar studies of human postmortem HIE are uncommon, so comparisons of our results to others are very limited. One study examined CR INs in the white matter of preterm infants and found a significant loss in white matter lesions in preterm infants compared to preterm infants not showing injury in the same white matter region [90]. In living patients, blink reflex abnormalities suggest abnormalities in INs after perinatal asphyxia [15].

### 4.4. TDP43 Proteinopathy Occurs in Neonatal HI Brain

We examined possible mechanisms underlying the loss of INs in the HI piglet brain that could involve DNA damage. TDP43 and XRCC1 function in DNA repair and control IN fate [30,51]. Both proteins regulate cell viability in general because the homozygous deletion of either is lethal for embryos [28,29]. Thus, we wondered if the suppression or inactivation of one or both proteins in somatic cells of the brain (e.g., INs) could be involved in acquired brain injury (Figure 14). In the sham piglet brain, we found ubiquitous, highly enriched immunoreactivity for TDP43 in virtually every cell in the neocortex. XRCC1 was also enriched in sham piglet brain neurons but had a delicate and subtle localization in the nucleus. TDP43 immunoreactivity was diminished and prominently redistributed from the nucleus in the HI piglet cerebral cortex, including its nuclear depletion in neurons and glial cells. The nuclear depletion of TDP43 is known to cause neurodegeneration in pigs [91]. The examination of TDP43 localization specifically in CR and PV INs in the somatosensory cortex of HI piglets revealed TDP43 mislocalization and aggregation within the nucleus and cytoplasm. These aggregates of TDP43 colocalized with aggregates of CR and PV, leading to the idea that CR and PV sequester TDP43. Follow-up co-immunoprecipitation showed that TDP43 interacted with CR and PV in HI piglets, but not sham, cerebral cortex. Dual labeling for these calcium-binding proteins and XRCC1 revealed a similar trapping of XRCC1 away from its normal nuclear localization. Because XRCC1 is a critical scaffold protein that functions in DNA-SSB repair [55], we evaluated INs for DNA-SSB accumulation using PANT. CR and PV INs in the neocortex accumulated DNA-SSBs after HI. While these biochemical and histological results suggest interactions between TDP43 or XRCC1 with CR and PV, it remains to be determined if these putative interactions are directly between two proteins or mediated by other proteins not yet identified here. For example, CR and TDP43 both physically interact with valosin-containing protein, and CR and XRCC1 interact with forkhead box D3 transcription factor (The Biological General Repository for Interaction Datasets, BioGRID|Database of Protein, Chemical, and Genetic Interactions), so these proteins could serve as intermediaries. Future experiments using mass spectroscopy on CR and PV and their co-precipitating proteins from piglet brains need to be performed. Cell and cell-free experiments using recombinant proteins would also be useful in this regard to strengthen our mechanistic claim regarding the toxic trapping of TDP43 and XRCC1 by CR and PV after they are subjected to nitrative or oxidative agents. These experiments would include CR and PV mutants with specific amino acid residues, such as tyrosines, tryptophans, and lysines, replaced with non-vulnerable amino acids by site-directed mutagenesis.

TDP43 and DNA repair pathologies in CR INs could also be downstream to abnormalities in intracellular Ca^2+^. Homologous recombination DNA repair is Ca^2+^-dependent [92]. Perturbations in nucleocytoplasmic Ca^2+^ shuttling stemming from abnormal CR function could affect DNA-DSB resolution. Moreover, elevated free cytosolic Ca^2+^ can affect TDP43 nucleoplasmic transport and its cytoplasmic aggregation and sequestration [93].

### 4.5. CR Is Nitrated in HI Piglet Cerebral Cortex

To understand the mechanisms for the CR and PV trapping of TDP3 and XRCC1, we considered aberrant post-translational modification (Figure 14). Modification by tyrosine nitration is known to cause anomalously altered, possibly toxic, properties in proteins, including aggregation [59,92,93]. Tyrosine nitration is a footprint for pathological mechanisms involving the nitric oxide (NO) pathway and the toxic mediator peroxynitrite [94,95]. The involvement of NO in the mechanisms of neonatal HIE has been suggested in patients [96] and pigs [74,97]. The protein sequence of CR is known to have tyrosine residues [98]. Swine and human CR have 12 tyrosine residues (~4.4% of amino acids) in its sequence (https://www.uniprot.org/uniprotkb/P22676/entry, accessed on 4 October 2024) and most are in the critical E–F hand Ca^2+^-binding motifs, which again could affect intracellular Ca^2+^ nucleocytoplasmic shuttling. We found CR to be nitrated after HI. However, PV in swine and human is different from CR because it has no tyrosine residues (https://www.uniprot.org/uniprotkb/P20472/entry, accessed on 4 October 2024) or tryptophan residues that can also be nitrated [99]. Instead, PV is highly enriched in lysine residues that are prone to carbonyl oxidative modification that can trigger protein aggregation [100] and, indeed, PV functions as a metal-dependent free radical scavenger in addition to its Ca^2+^ shuttling function [101]. Protein carbonyl levels are known to be increased subacutely in the cerebral cortex of HI piglets [102].

### 4.6. IN Cell Death in Neonatal HIE Can Be Called Aggreosis

We found that CR and PV INs degenerate, die, and are reduced in number in the neocortex of piglets and humans after neonatal HI. Their cell death has features of protein aggregation, TDP43 and XRCC1 mislocalization and sequestration, DNA damage accumulation, and attrition. The nuclear attrition was not like the karyorrhexis of ischemic-necrosis, seen in most HI-NT piglet cortical pyramidal neurons, or the chromatin clumping of apoptosis [85,103]. There were unremarkable cytoplasmic swelling or vacuolar changes indicative of loss of volume control or structural changes in mitochondria reminiscent of ischemic–necrosis or necroptosis [104]. There was no enhanced granularity or apparent lysosomal accumulation suggestive of autophagy. The distinguishing features of this IN cell death was TDP43 and XRCC1 exclusion from the nucleus and cytoplasmic aggregation and DNA strand break accumulation. We suggest the name “aggreosis” be given to this form of attritional, protein aggregation that is a prominent, non-apoptotic form of cell death seen in INs in neonatal HIE because it does not seem to match existing structural classifications and nomenclature (Figure 14).

## 5. Conclusions

IN degeneration might be linked to seizure development in neonatal HIE. Neonatal piglets received HI and subacute recoveries with NT or overnight HT, or they received sham procedures with NT or overnight HT. Piglets were euthanized at varying times with time-matched shams at 29 h–7 days. Naïve piglets were additional controls. Piglet brains were assessed using immunohistochemistry (single- and double-label) for INs markers CR, PV, VIP, and for the DNA repair proteins TDP43 and XRCC1. Other assessments were DNA fragmentation, co-immunoprecipitation, and immunoblotting. Postmortem human neonatal HIE and non-HIE brains were assessed also. Layer 2/3 CR INs and layer 4 PV INs were severely depleted in HI piglets without HT. VIP INs were not reduced in number in HI piglets. HT protected the neocortex globally and partially rescued the CR INs after HI. CR and PV formed nuclear and cytoplasmic inclusions colocalizing with TDP43 and XRCC1; co-immunoprecipitation verified interactions among these proteins as early as 29 h after HI. CR and PV INs accumulated DNA single- and double-strand breaks and died in a process identified as aggreosis. CR and PV INs were similarly depleted in human HIE neocortex compared to non-HIE neocortex. Loss of INs strongly correlated with seizure burden, but this does not prove causality. IN vulnerabilities appear to be instructed by their intrinsic calcium-binding protein that traps via protein–protein interaction with XRCC1 and TDP43. Their loss of function could cause faulty DNA repair and DNA damage accumulation or the presence of specific proteins containing “poison” cryptic exon-encoded sequences that drive IN cell death, seizures, and global neocortical damage in neonatal HIE. Studies of potential TDP43 loss of function need to be performed, including those addressing the possible accumulation of toxic cryptic exon-containing proteins in INs after neonatal HI.

## Data Availability

The original contributions presented in this study are included in the article/Supplementary Materials. Further inquiries can be directed to the Lee J. Martin.

## Supplementary Materials

The following supporting information can be downloaded at: https://www.mdpi.com/article/10.3390/biom16050621/s1, Figure S1. Electroencephalogram (EEG) patterns of recovery after resuscitation in 7–10-day-old piglets. (A) Early stage discontinuous: isoelectric >50% of the epoch, alternating with low voltage (5–10 μV) slow activity (1–5 Hz). (B) Intermediate discontinuous: isoelectric 10–50% of the epoch, alternating with low voltage (5–10 μV) slow activity (1–5 Hz). (C) Late discontinuous: regular brief intervals (up to 10% of epoch) of isoelectric record alternating with disorganized bursts of medium-to-high voltage (20–70 μV) mostly slow activity. (D) Emergence of early continuous: mostly low voltage (5–10 μV) slow and medium frequencies (1–8 Hz), no sleep–wake shifts. (E) Intermediate continuous: mix of voltages (10–50 μ V), greater mix of frequencies (3–16 Hz), no sleep–wake shifts. (F) Fully continuous: like E, with addition of spontaneous sleep–wake shifts, depicted as /*/. Figure S2. Summary showframe of experimental design for neonatal HIE in 2–3-day-old piglets. Figure S3. Examples of post-acquisition processed cEEG for piglets treated with sham or HI procedures and normothermic (NT) of hypothermic (HT) recoveries for 29 h. Supplementary Videos repository is https://doi.org/10.5281/zenodo.18549427 (accessed on 9 February 2026)): Video S1. HI-HT and sham-NT piglets at ~28 h after extubation and ~52 h after the insult. Surgical sites for cEEG array installation can be seen by sutures with telemetry antenna exiting posteriorly. The dish for milk is seen at the bottom. The HI-HT piglet is in the cow-pattern wrap (ear tag AF331). The sham-piglet is in the black wrap (ear tag AF330). The HI-HT piglet is somnolent and tremulous. Video pans to real time cEEG for this piglet (right panel) showing normal body temperature at 37.5 °C. From top to bottom, the leads are recording from somatosensory cortex right anterior, right posterior, left anterior, and left posterior. cEEG shows coinciding seizures in 3 of 4 leads. Sham-NT piglet has a body temperature of 38.1 °C. The sham-NT piglet shows arousal and activity. Corresponding movement is detected in the cEEG (left panel). Video S2. HI-NT and sham-HT piglets at ~ 9 h after extubation and ~45 h after the insult. The HI-NT piglet is in a pink-white checkered wrap. The sham-HT piglet is in the blue wrap. Both piglets are somnolent, but the HI-NT piglet was recently awake and drinking milk as indicated by the milk-beard. The sham-HT piglet is sleeping soundly with no evidence of clinical seizures. In contrast, the HI-NT piglet is showing clinical seizure activity with clonic–tonic forelimb muscles and jerking movements. The hindlegs appear to have normal tone. The video pans to the real time cEEG for this piglet (right panel) showing, from top to bottom, recording of somatosensory cortex right anterior (G1), right posterior (G2), left anterior (G3), and left posterior (G4). cEEG shows notable seizures in the right anterior somatosensory cortex. The downward deflections are telemetry dropout. Video S3. HI-NT and sham-HT piglets ~5 min after Video S2 recording. The HI-NT piglet is in a pink-white checkered wrap. The Sham-HT piglet is in the blue wrap and is sleeping soundly with a body temperature of 36.9 °C. In contrast, the HI-NT piglet shows fictive running and spasticity of the forelegs that remain with passive manipulation of the limbs by an examiner (LJM). The hindlegs appear normal in their responses to passive stretch. The video pans to the real time cEEG for this piglet (right panel) showing, from top to bottom, recording of somatosensory cortex right anterior (G1), right posterior (G2), left anterior (G3), and left posterior (G4). cEEG shows brief seizures in 3 of 4 leads. Video S4. HI-NT and sham-HT piglets at ~ 12 h after extubation and ~48 h after the insult. These piglets are the same as in Videos 2 and 3. The sham-HT piglet is in the blue wrap and is sleeping soundly. The HI-NT piglet is in the pink-white checkered wrap (ear tag 357) and shows transient orofacial movements and fictive running. The video pans to the real time body temperature (37.6 °C) and cEEG for this piglet (right panel) showing, from top to bottom, recording of somatosensory cortex right anterior (G1), right posterior (G2), left anterior (G3), and left posterior (G4). cEEG shows notable seizure activity in the right anterior somatosensory cortex. Video S5. HI-HT piglet (ear tag AF421) 6 days after rewarming. This piglet is showing prolonged clonic–tonic seizures in apparent status epilepticus. The piglet was euthanized later that day. Video S6. Sham-HT piglet (ear tag 389) ~20 h after rewarming and ~46 h after the sham procedure. Piglet is seizing as indicated by the robust fictive running and whole-body jerking movements. Table S1: Postmortem Human Cases Used.

## Abbreviations

The following abbreviations are used in this manuscript: CR, calretinin; CV, cresyl violet; DNA-DSB, double-strand break; EEG, electroencephalogram; cEEG, continuous EEG; H&E, hematoxylin & eosin; HT, hypothermia (hypothermic); HI, hypoxic-ischemic; HIE, hypoxic-ischemic encephalopathy; IN, interneuron; NT, normothermia (normothermic); PV, parvalbumin; DNA-SSB, single-strand break; TDP43, TAR DNA binding protein of 43 kDa; VIP, vasoactive intestinal peptide; WB, western blot; XRCC1, X-ray repair cross complementing-1.
