# Supplementary material for: Calretinin and Parvalbumin Trapping of TDP43 and XRCC1 Instructs Neocortical Interneuron Death in Neonatal Hypoxic-Ischemic Encephalopathy
Source: Biomolecules. 2026 Apr 22;16(5):621. doi: 10.3390/biom16050621 (PMC13204630; doi:10.3390/biom16050621)

Journal name: Biomolecules

Manuscript ID: biomolecules-4173147

Type of manuscript: Article

Title: Calretinin and Parvalbumin Trapping of TDP43 and XRCC1 Instructs Neocortical Interneuron Death in Neonatal Hypoxic-Ischemic Encephalopathy

Authors: Lee J. Martin *, Rebecca N Ichord, Caitlin E O'Brien, Sophie Yohannan, Danay Fernandez, Annalise Garrido, Naya Amauri, Dongseok Park, Jordan Benderoth, Jennifer K Lee

Supplemental Data Full-Length Western Blots Used in Figures- Lee Martin

Figure 4 A

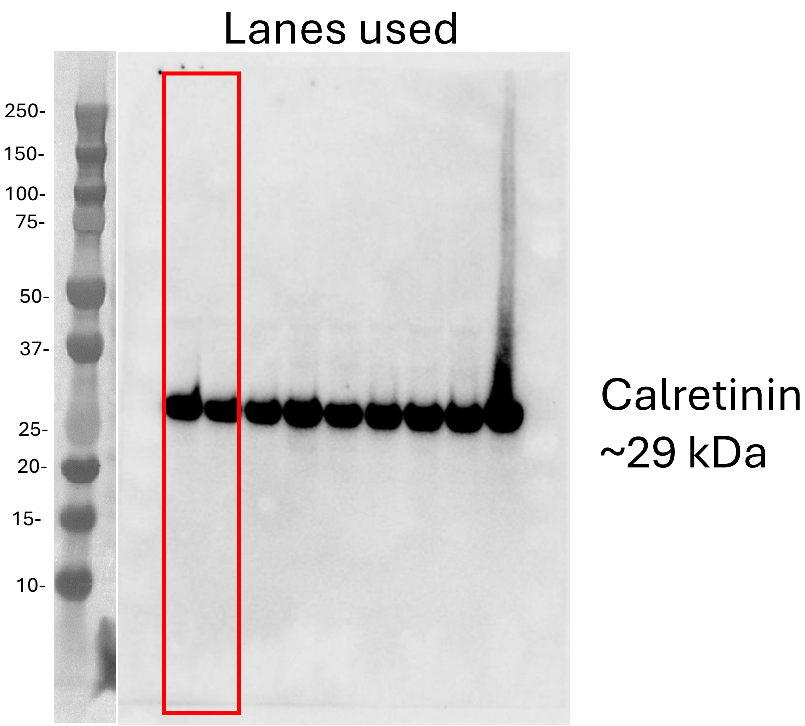

Figure 4B

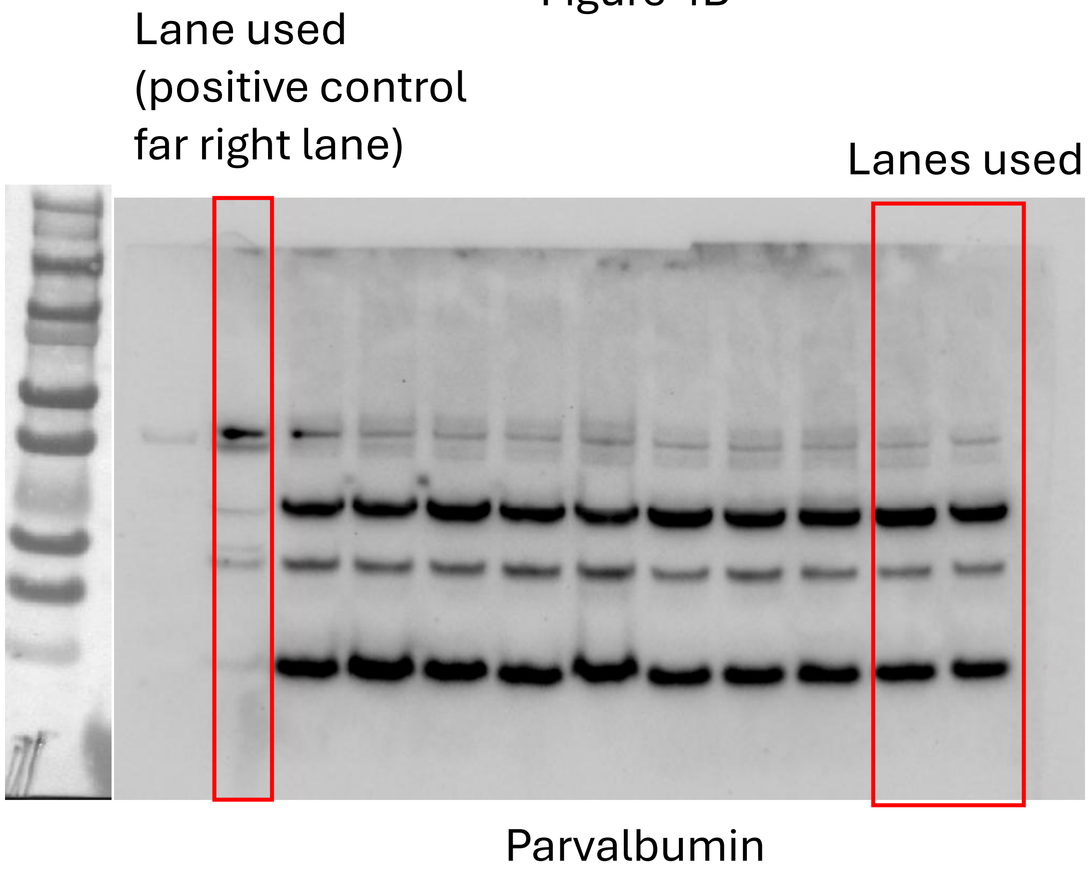

Figure 8A VIP western blot

Lanes used

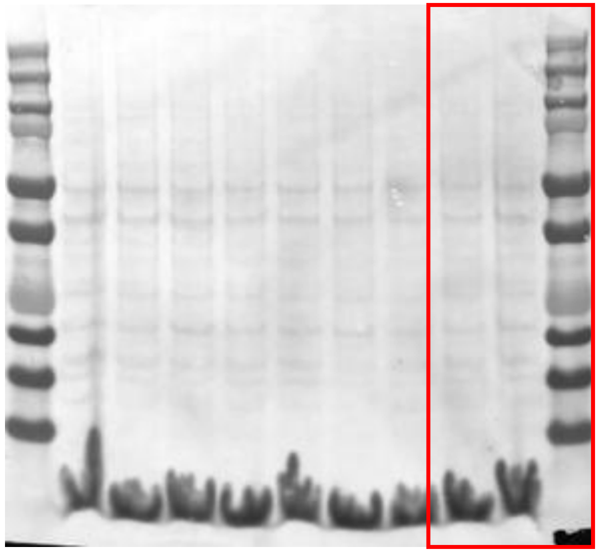

Ponceau

Lanes used

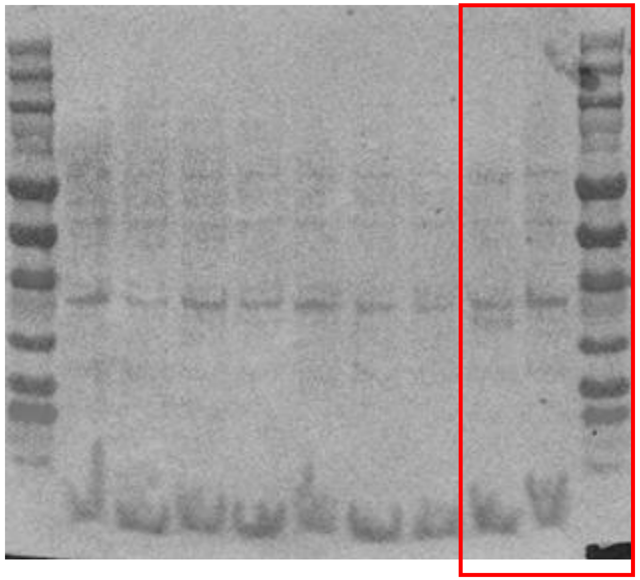

VIP

Figure 9A

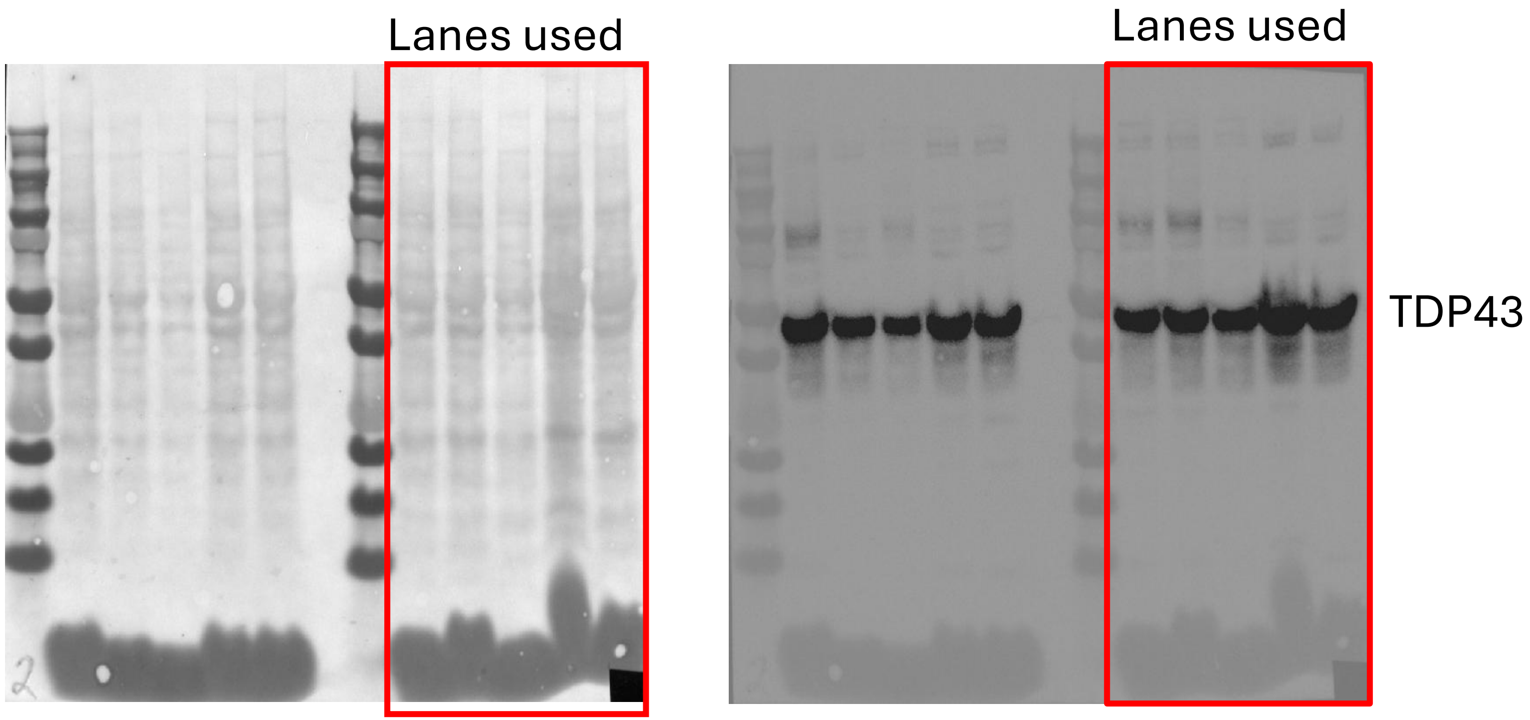

Ponceau

Figure 10F

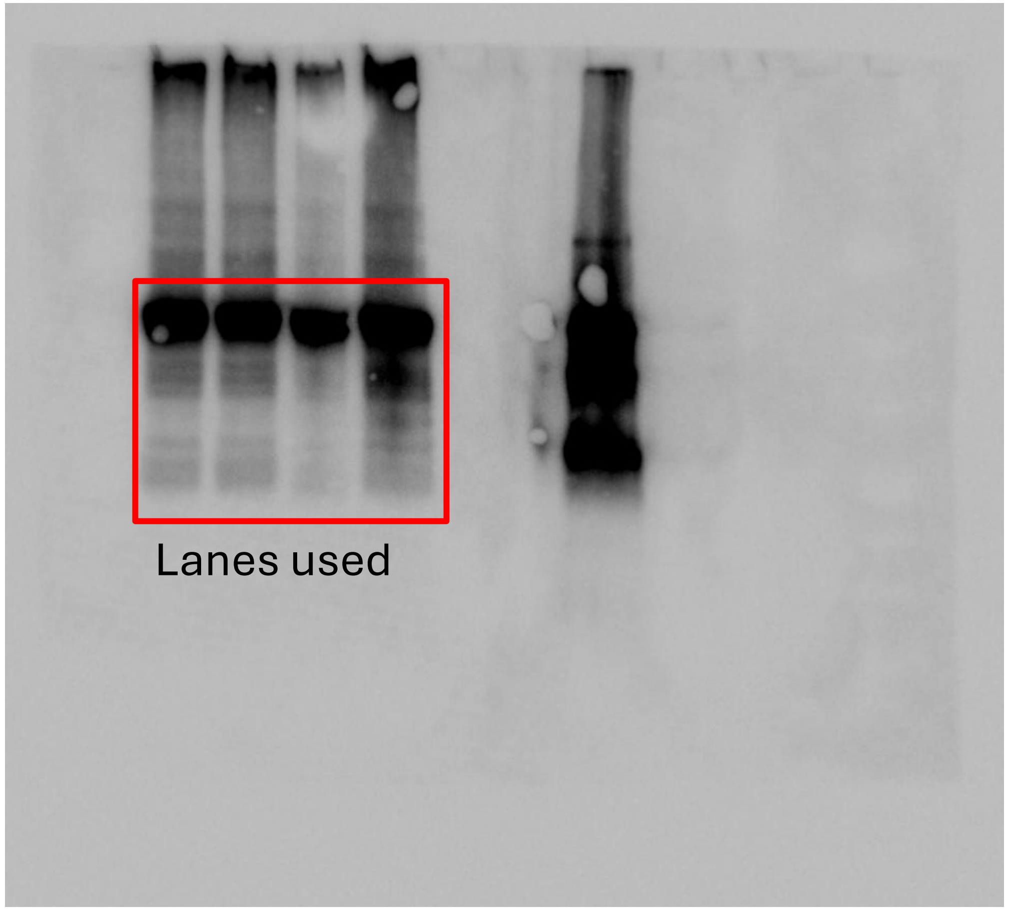

Figure 10G

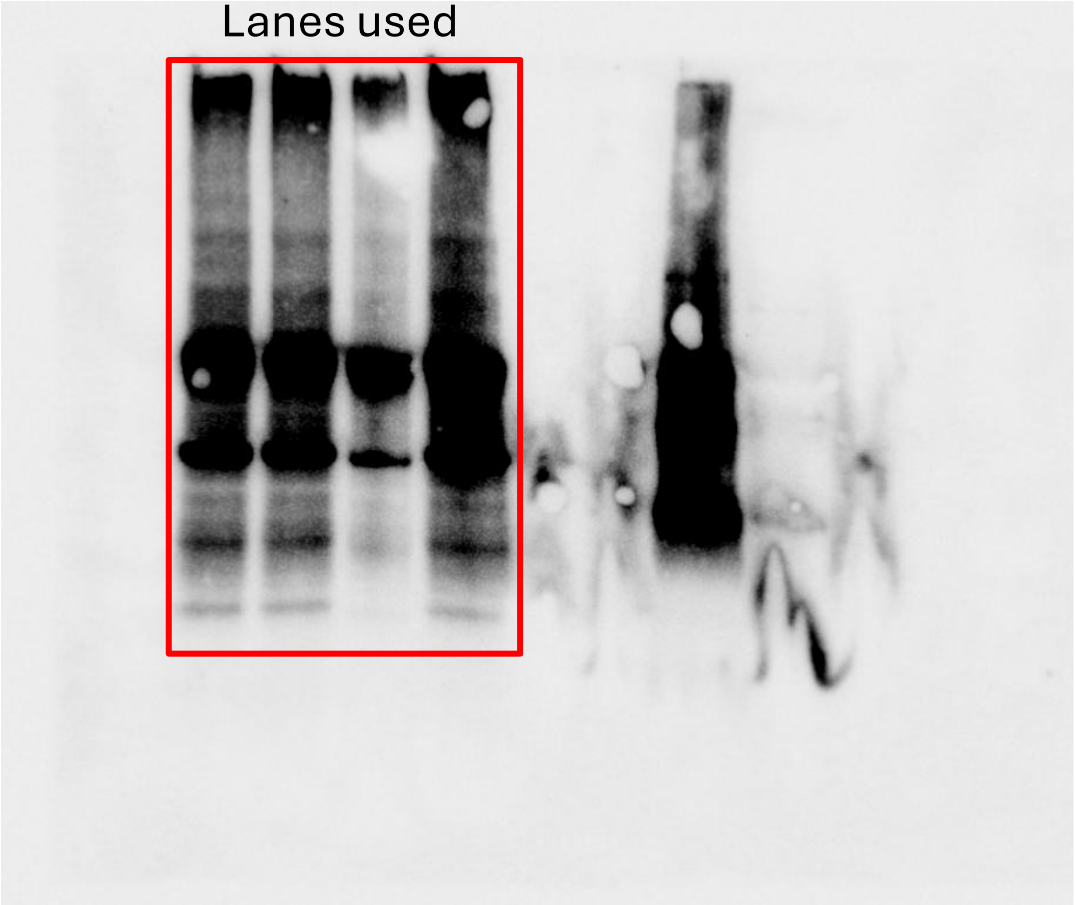

Figure 11F

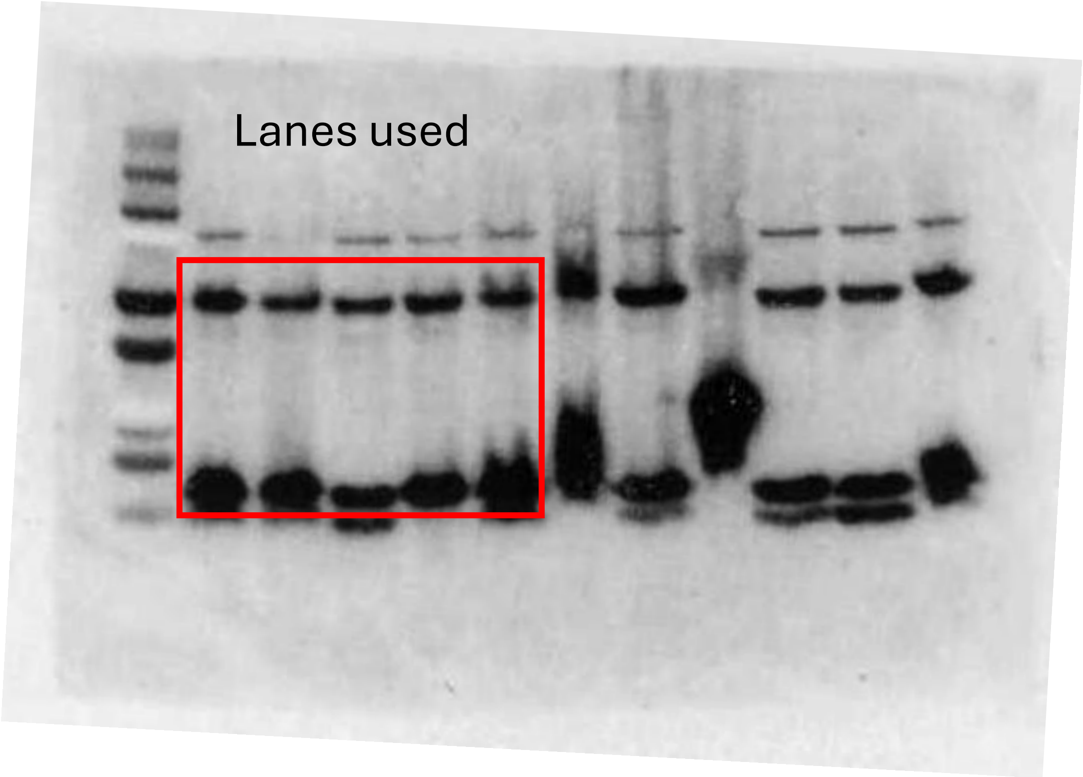

Negative Control Western Blots: Antibody Validation

Mouse IgG Non-Immune IgG Isotype

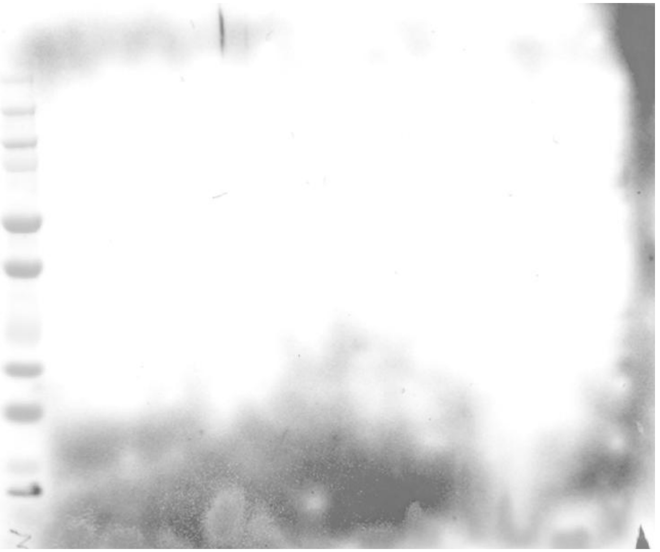

Ponceau of same membrane above

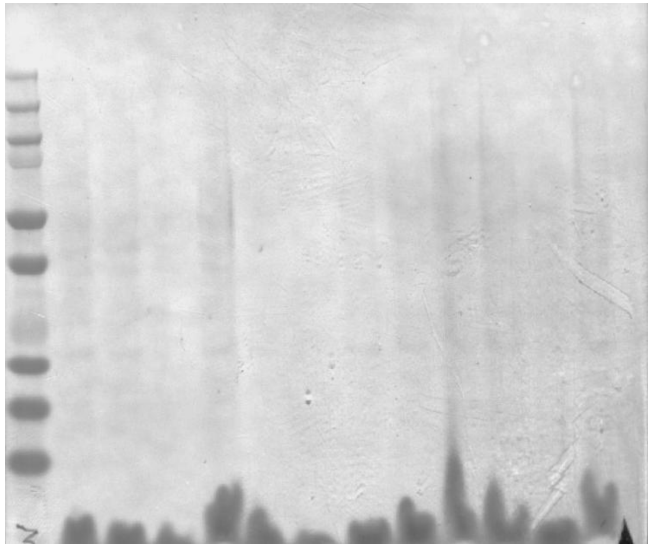

Rabbit IgG Non-Immune IgG Isotype

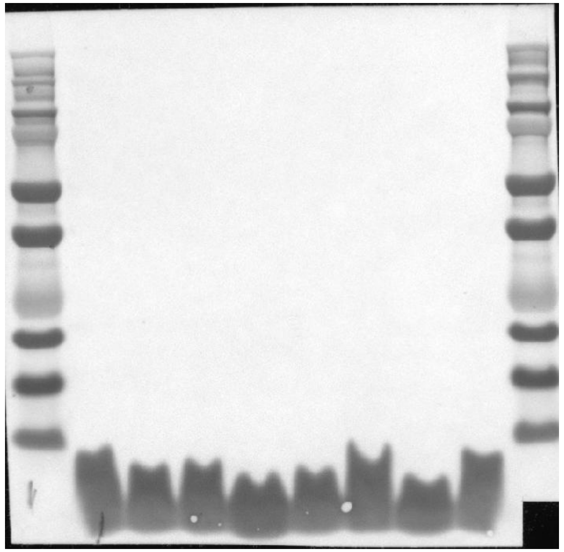

Ponceau of same membrane above

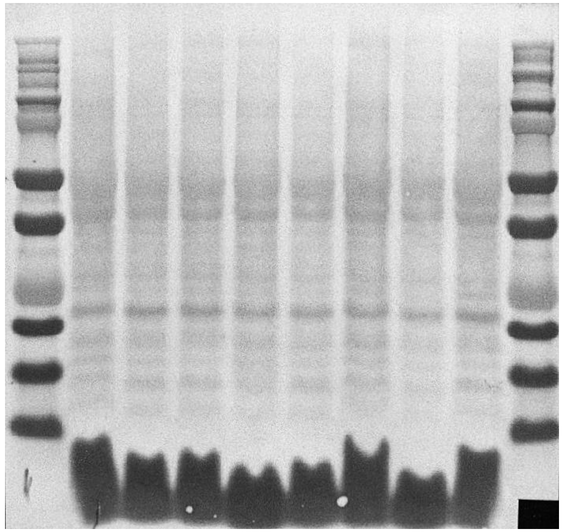

Supplement: Supplementary file 1 [file biomolecules-16-00621-s001.zip › biomolecules-4173147 Full Length Western blots.pdf]
