# Supplementary material for: Calretinin and Parvalbumin Trapping of TDP43 and XRCC1 Instructs Neocortical Interneuron Death in Neonatal Hypoxic-Ischemic Encephalopathy
Source: Biomolecules. 2026 Apr 22;16(5):621. doi: 10.3390/biom16050621 (PMC13204630; doi:10.3390/biom16050621)
Supplement: Supplementary file 1 [file biomolecules-16-00621-s001.zip › Supplementary Figure Legends.pdf]

Supplementary Figure 1. Electroencephalogram (EEG) patterns of recovery after resuscitation in 7–10-day old piglets. (A) Early stage discontinuous: isoelectric >50% of the epoch, alternating with low voltage (5-10 μV) slow activity (1-5 Hz). (B) Intermediate discontinuous: isoelectric 10-50% of the epoch, alternating with low voltage (5-10 μV) slow activity (1-5 Hz). (C) Late discontinuous: regular brief intervals (up to 10% of epoch) of isoelectric record alternating with disorganized bursts of medium-to-high voltage (20-70 μV) mostly slow activity. (D) Emergence of early continuous: mostly low voltage (5-10 μV) slow and medium frequencies (1-8 Hz), no sleep-wake shifts. (E) Intermediate continuous: mix of voltages (10-50 μ V), greater mix of frequencies (3-16 Hz), no sleep-wake shifts. (F) Fully continuous: like E, with addition of spontaneous sleep-wake shifts, depicted as /*/.

Supplementary Figure 2. Summary diagram of experimental design for neonatal HIE in 2–3-day old piglets.

Supplementary Figure 3. Examples of post-acquisition processed cEEG for piglets treated with sham or HI procedures and normothermic (NT) of hypothermic (HT) recoveries for 29 hours.
