# Supplementary material for: Calretinin and Parvalbumin Trapping of TDP43 and XRCC1 Instructs Neocortical Interneuron Death in Neonatal Hypoxic-Ischemic Encephalopathy
Source: Biomolecules. 2026 Apr 22;16(5):621. doi: 10.3390/biom16050621 (PMC13204630; doi:10.3390/biom16050621)
Supplement: Supplementary file 1 [file biomolecules-16-00621-s001.zip › Table of Human Postmortem Cases Used.pdf]

Case Identifier	Age at birth (weeks)	Clinical setting	Therapeutic Hypothermia	Age (at death)	Postmortem delay (hours)
A15-7	36 (37)	Fetal deceleration and emergency C-section. Resuscitation involving chest compressions, gas of 6.9, and clinical seizures	Yes	7 days	24
A16-13	41.6 (42.9)	Shoulder dystocia; prolonged ruptured membranes	Yes	9 days	12
A16-30	34 (34.3)	Car collision, placental abruption, emergency C-section, DIC in newborn, neonatal respiratory failure	Yes	2 days	144
A17-1	39.3 (39.9)	Rupture of membranes, C-section, required chest compressions, cord gas of 6.9	Yes	4 days	12
A17-3	38 (38.4)	Uncontrolled insulin-dependent diabetes (mother), emergency C-section, perinatal asphyxia, chest compressions	Yes	3 days	24
A17-14	24 (48.6)	Chronic lung disease with secondary pulmonary hypertension, hypoxemic respiratory failure, worsening hypotension	0 (missed therapeutic time window)	172 days	25
A18-2	35.3 (35.9)	Diamniotic dichorionic twins, premature rupture of membranes, emergency C-section, Traumatic delivery (cephalohematoma, focal subdural hemorrhage, subarachnoid hemorrhages), chest compressions	Yes	4 days	16
A18-3	39.9 (40.4)	Secondary apnea, respiratory failure, multiorgan failure, possible septic shock	Yes	4 days	24
A18-17	34.3 (36.4)	HIV+ mother via emergency C section for fetal deceleration for systole, chest compressions	0 (missed therapeutic time window)	15 days	48
A18-28	40.3 (40.4)	Non-reassuring fetal heart rate, acute phlebitis of umbilical cord, acute chorioamnionitis of membranes, chorionic plate had acute subchorionitis, tight nuchal chord, placental SGA	Yes	1 day	408
4314	38	Respiratory insufficiency, anoxic encephalopathy	No	4 days	21

667	38	Acute cardiac arrhythmia-arrest (Non-HIE control)	No	353 days	13
828	36	Meconium aspiration, seizure disorder	No	90 days	10
731	36	Severe birth anoxia, anoxic encephalopathy, seizure disorder	No	360 days	14
A54802	39	Rupture of membranes, cardiorespiratory arrest	Yes	4 days	24
A56447	35	Non-reassuring fetal statue, C-section, immediate apnea, respiratory arrest. Anoxic encephalopathy	No (missed therapeutic time window)	5 days	24
A54854	34.5	Maternal gestational diabetes, late decelerations during cerclage removal, emergency C-section	Yes	1 day	22
1047	unavailable	Non-HIE	NA	7 years	24
993	unavailable	Non-HIE	NA	1 year	12
783	unavailable	Non-HIE	NA	8 years	19
875	unavailable	Non-HIE	NA	3 years	10
1176	unavailable	Non-HIE	NA	20 years	6
