# Supplementary figures and images for: Calretinin and Parvalbumin Trapping of TDP43 and XRCC1 Instructs Neocortical Interneuron Death in Neonatal Hypoxic-Ischemic Encephalopathy
Source: Biomolecules. 2026 Apr 22;16(5):621. doi: 10.3390/biom16050621 (PMC13204630; doi:10.3390/biom16050621)

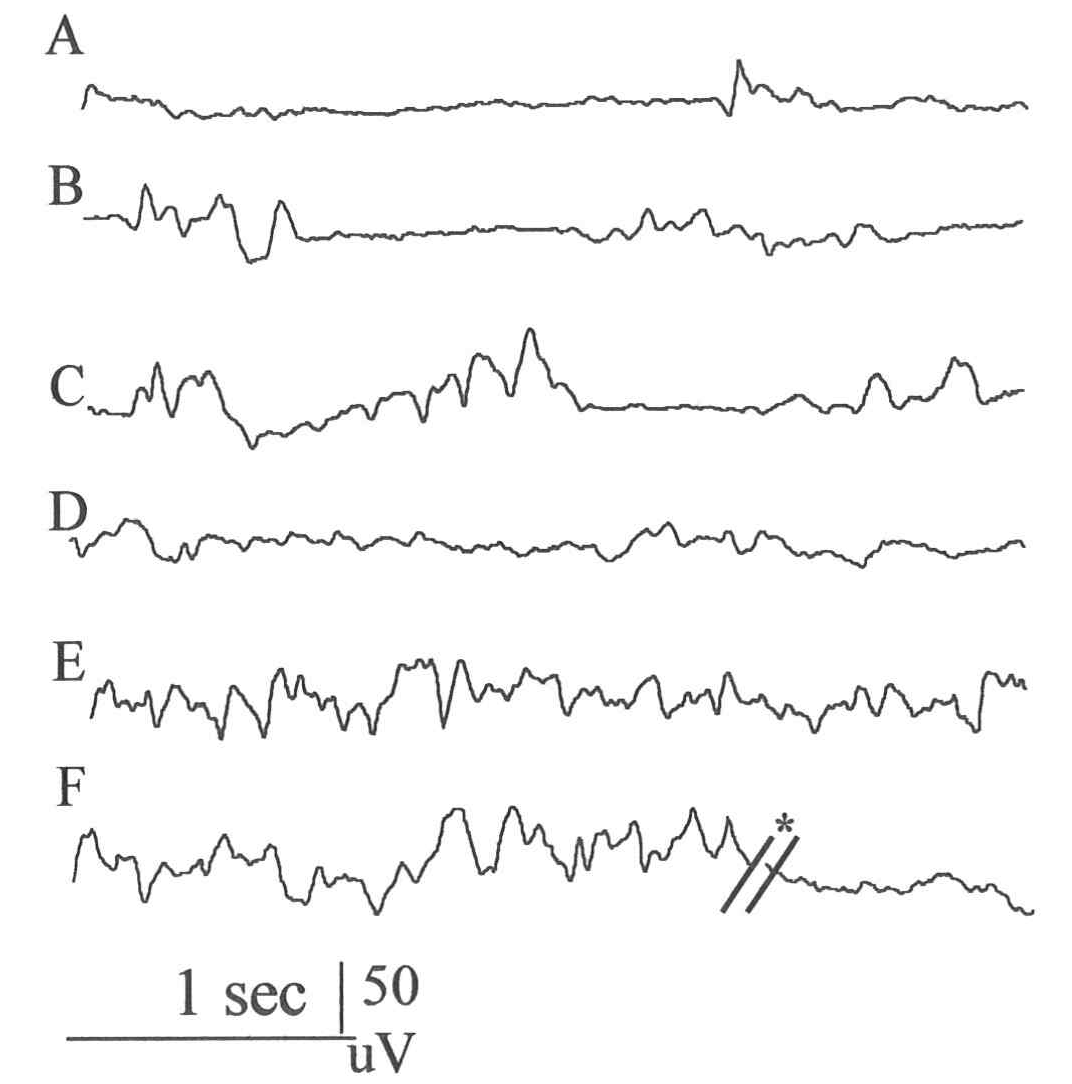

Supplement: Supplementary file 1 [file biomolecules-16-00621-s001.zip › Figure S1.tif]

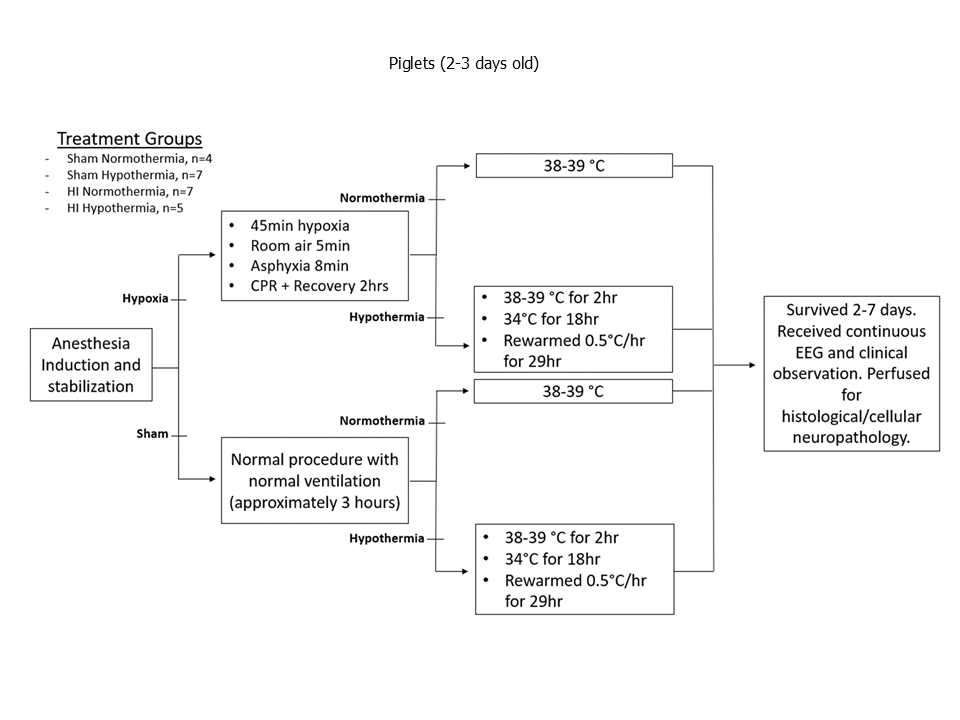

Supplement: Supplementary file 1 [file biomolecules-16-00621-s001.zip › Figure S2 Revised.tif]

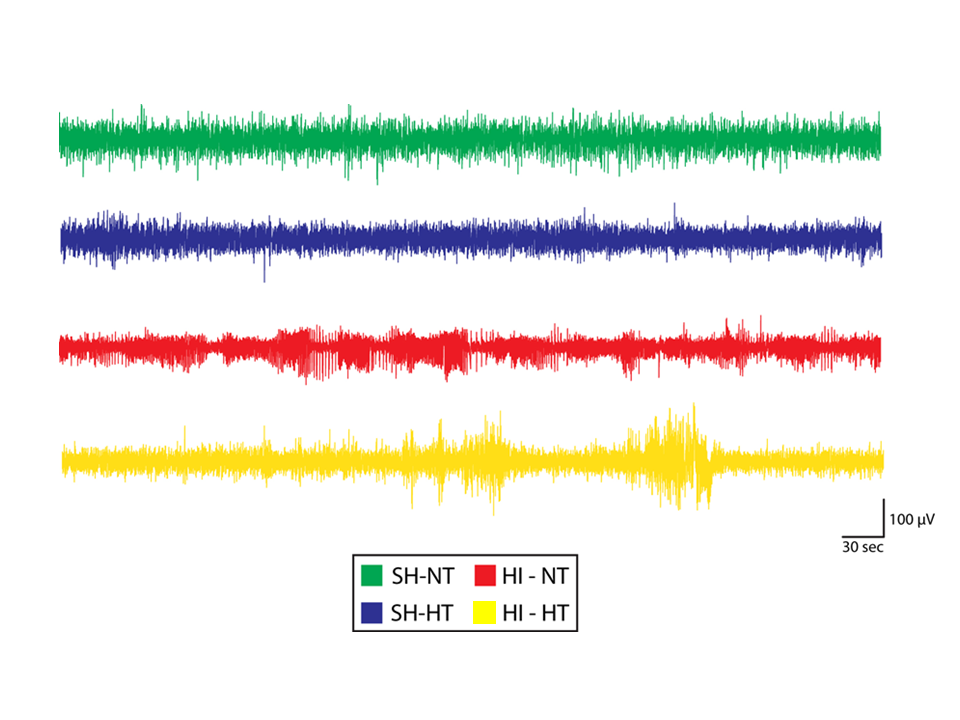

Supplement: Supplementary file 1 [file biomolecules-16-00621-s001.zip › Figure S3 Revised.tif]
